# Associations between activity patterns and cardio-metabolic risk factors in children and adolescents: A systematic review

**DOI:** 10.1371/journal.pone.0201947

**Published:** 2018-08-16

**Authors:** Simone J. J. M. Verswijveren, Karen E. Lamb, Lisa A. Bell, Anna Timperio, Jo Salmon, Nicola D. Ridgers

**Affiliations:** 1 Institute for Physical Activity and Nutrition (IPAN), School of Exercise and Nutrition Sciences, Deakin University, Geelong, Australia; 2 Barwon Health, Child Health Research Unit, Geelong, Victoria, Australia; Vanderbilt University, UNITED STATES

## Abstract

**Introduction:**

Total volumes of physical activity and sedentary behaviour have been associated with cardio-metabolic risk profiles; however, little research has examined whether patterns of activity (e.g., prolonged bouts, frequency of breaks in sitting) impact cardio-metabolic risk. The aim of this review was to synthesise the evidence concerning associations between activity patterns and cardio-metabolic risk factors in children and adolescents aged 5–19 years.

**Materials and methods:**

A systematic search of seven databases was completed in October 2017. Included studies were required to report associations between objectively-measured activity patterns and cardio-metabolic risk factors in children and/or adolescents, and be published between 1980 and 2017. At least two researchers independently screened each study, extracted data, and undertook risk of bias assessments.

**Results:**

From the 15,947 articles identified, 29 were included in this review. Twenty-four studies were observational (cross-sectional and/or longitudinal); five were experimental. Ten studies examined physical activity patterns, whilst 19 studies examined sedentary patterns. Only one study examined both physical activity and sedentary time patterns. Considerable variation in definitions of activity patterns made it impossible to identify which activity patterns were most beneficial to children’s and adolescents’ cardio-metabolic health. However, potential insights and current research gaps were identified.

**Discussion and conclusion:**

A consensus on how to define activity patterns is needed in order to determine which activity patterns are associated with children’s and adolescents’ cardio-metabolic risk. This will inform future research on the impact of activity patterns on children’s and adolescents’ short- and longer-term health.

## Introduction

Children’s and adolescents’ overweight and obesity rates have increased over the past four decades and are at unprecedented levels in the population [1]. This increasing prevalence of obesity has been accompanied by a rise in the prevalence of type 2 diabetes and other cardio-metabolic disturbances, such as impaired glucose and lipid accumulation [2]. Physical inactivity, defined as an insufficient level of physical activity to meet present physical activity recommendations [3], is a key contributor to this poor health status [4, 5]. Low levels of physical activity (i.e. any bodily movement produced by skeletal muscles that results in energy expenditure [6]) are detrimentally associated with cardio-metabolic risk factors in school-aged children and adolescents [7–12]. Excessive participation in sedentary behaviour (i.e. any waking behaviour characterised by an energy expenditure ≤1.5 metabolic equivalents (METs), while in a sitting, reclining or lying posture [3]), has also been detrimentally associated with cardio-metabolic risk factors in children and adolescents [13–15]. Research has shown that cardio-metabolic risk factors and activity levels track into adulthood [1, 16, 17].

The total daily volume of both physical activity and sedentary time is often considered a predictor of health, though it is unclear whether there are differential effects on health depending on how these are accumulated by children and adolescents [11, 18, 19]. Activity is accumulated in bouts, which vary in frequency, intensity, and duration (adapted from [3]), yet the traditional focus on volume ignores such patterns of accumulation. In adults, sustained (defined as bouts lasting ≥10 min) and short (<10 min) bouts of moderate-to-vigorous physical activity (MVPA) are associated with a lower body mass index (BMI) and waist circumference [20], while breaks in sedentary time (adjusted for total sedentary time) are beneficially associated with triglycerides [21], adiposity, and glucose metabolism [21, 22]. This suggests that in adults, it is not only the total volume of physical activity and sedentary time that is important for health, but also the way in which these are accumulated.

Despite adolescents accumulating less physical activity than children [23], research has shown that children and adolescents accumulate their physical activity in short, sporadic bouts [24, 25]. Objective monitoring devices, such as accelerometers, enable researchers to capture specific activity behaviours that are date and time stamped. As such, it is possible to explore children and adolescents’ activity levels in detail (e.g. bouts of activity) [26]. However, there is still very little scientific understanding regarding whether “activity patterns” (that is, the way in which children and adolescents accrue their sedentary time and physical activity) are similarly important for children’s and adolescents’ health. Activity patterns such as prolonged bouts of sitting are characterised by the absence of skeletal muscle contractile activity, particularly in the lower limbs and postural muscles. It is suggested that lower contractile activity is associated with reduced blood flow and efficiency of many of the body’s regulatory processes, such as the transport of blood glucose from the circulation into the muscle [27]. Just assessing total volume of activity will fail to capture how activity patterns relate to cardio-metabolic health in children and adolescents. Assessing activity patterns will provide insights into which activity patterns could be considered in the design of strategies to optimise health and in the future refinement of public health guidelines. This is particularly pertinent as current efforts to change overall activity levels (i.e. focus on total volume) have had limited effectiveness [28].

To date, several reviews have reported associations between activity patterns and health and well-being outcomes among children and adolescents [11, 15, 18]. The focus of these reviews was primarily on either the total volume of physical activity [15, 18] or sedentary time [11], and included many health outcomes, such as cognition and academic achievement. For example, Cliff and colleagues’ review and Carson and colleagues’ narrative review both focused on sedentary time and patterns [15, 18]. These included only objective [18] or both objective and subjective measures, respectively [15]. In comparison, Poitras and colleagues conducted a narrative synthesis, which only focused on physical activity. While these reviews provided useful information in many areas of health and well-being, none examined in specific detail the associations between activity patterns across the entire activity spectrum and health. Because patterns of both physical activity and sedentary behaviour occur on an activity spectrum [19], and are potentially important for health [21, 22], volumes and/or patterns of each behaviour should be reported simultaneously [19]. In addition, since these reviews were conducted (i.e., November 2015, the latest), valuable new research might have been published. Consequently, the aim of this review was to examine associations between activity patterns across the activity spectrum (i.e., all waking behaviours, including sedentary behaviour and low and high intensity activity patterns) and cardio-metabolic risk factors in children and adolescents aged 5–19 years.

## Materials and methods

This systematic review was conducted in accordance with the Preferred Reporting Items for Systematic Reviews and Meta-Analyses (PRISMA) statement [29]. It was registered with the PROSPERO International Prospective Register of Systematic Reviews (number CRD42016046764). The PICO principle (P—population; I—intervention/exposure; C—comparison (N/A); O—outcome) was utilised to develop a search strategy based on the aim of the review [30].

### Searches

A systematic literature search of EBSCOhost (Academic Search Complete, Education Source, ERIC, Global Health, MEDLINE Complete, SPORTDiscus) and Scopus databases was conducted in October 2017. The PICO-based search strategies for the databases included the following key words in three main areas: 1) Population “children and adolescents” (e.g., youth); AND 2) Intervention/exposure “activity” (e.g., physical activity, sedentary behaviour) AND “activity patterns” (e.g., bouts, transitions, breaks) AND “objective measurement” (e.g., accelerometer, inclinometer); AND 3) Outcome “cardio-metabolic health” (e.g., BMI, blood pressure). The EBSCOhost search strategy is shown in S1 Table as an example. Articles were extracted and imported into EndNote X7.5 (Thomson Reuters, New York, USA).

### Study inclusion and exclusion criteria

Studies were eligible for inclusion in this review if they: a) used a quantitative research design (e.g., cross-sectional, randomised controlled trial); b) included participants aged 5–19 years (i.e. likely to be attending primary or secondary school); c) reported associations between activity patterns of any intensity (i.e., sedentary, light, moderate, vigorous, very hard) with cardio-metabolic risk factors (i.e., adiposity, blood lipids, inflammatory biomarkers, endothelial function biomarkers, blood glucose, vascular health, fitness, or summary cardio-metabolic scores); d) objectively measured activity patterns (e.g., accelerometer); e) were published between January 1980 and October 2017; and f) were published in peer-reviewed journals in English or Dutch. Studies that were an advanced publication ahead of print and had a unique digital object identifier (DOI) were eligible for inclusion. To form a dataset representative of the targeted population (i.e., children and adolescents 5–19 years old), studies were excluded if they specifically focused on special/clinical populations (e.g., elite athletes, type 1 diabetes, being treated for obesity/overweight, children and adolescents chosen for family history of specific diseases).

### Data extraction strategy

Titles and abstracts were screened by three independent reviewers (LAB, SEC and SJJMV), and full text copies were obtained for all articles that met the initial screening criteria or where the appropriateness of the article was unclear. Three reviewers (NDR, SEC and SJJMV) then independently reviewed the full text of these papers for eligibility for inclusion. Inconsistencies between reviewers were discussed and resolved, and if a consensus could not be reached, the papers were discussed by all authors. Reference lists of included articles were also reviewed to identify any additional studies. Data were extracted by one reviewer (SJJMV) and checked and verified for 15% of articles by another reviewer (LAB) for consistency. Results were consistent and both reviewers agreed on the direction of associations. For the remaining articles, if the reviewer (SJJMV) was uncertain about the interpretation of included results, these were discussed amongst the team members. If more clarification was needed, the authors were contacted via email for clarification. Data extraction was undertaken using a standardised form which included all relevant PICO components; Population (e.g., sample size, sex, BMI, age, and school grade), Intervention/exposure (i.e., activity pattern), and Outcome (i.e., cardio-metabolic risk factors). Information on study characteristics (e.g., study design, type of objective measurement) and statistical methods (e.g., correlations, regression) was also extracted.

### Risk of bias assessment

Information on risk of bias (ROB) for individual articles was extracted by two reviewers (LAB and SJJMV) using a tool based on items of the ‘EPHPP Quality Assessment Tool for Quantitative Studies’ [31] (see S1 Text). Four methodological components were assessed in eight items: 1) Selection bias (e.g., sample representativeness); 2) Confounders (e.g., control for relevant confounders); 3) Data collection methods (e.g., whether the methods were reported to be valid and reliable); and 4) Withdrawals and drop-outs (e.g., number of withdrawals) [31]. Each component was given a quality rating of weak, moderate or strong, based on the accompanying instructions for the tool [31]. Studies that received no weak components on the four ROB components were classified as ‘low ROB’. Studies that received one weak rating were classified as ‘medium ROB’, while studies that received two or more weak ratings were classified as ‘high ROB’ [31]. Results were consistent between the reviewers and any disagreements were discussed until a decision was made.

### Data synthesis

Any type of activity pattern (e.g. sporadic and prolonged bouts, breaks) of all intensities (i.e., ranging from sedentary to very hard physical activity [VHPA]) was included if it was examined in relation to cardio-metabolic risk factors. According to STROBE (i.e., STrengthening the Reporting of OBservational studies in Epidemiology) guidelines [32], both unadjusted and fully-adjusted models should be presented in epidemiological studies. However, most studies [33–49] (17/29; 59%) included in this review only presented adjusted models (including adjustments for BMI, total MVPA, and age, for example), and six studies (21%) [50–55] presented only unadjusted models. The remaining six studies (21%) [14, 56–60] presented both. Fourteen of the 29 included studies (48%) adjusted for either total MVPA [14, 33, 36, 38, 40–42, 58], sedentary time [60], or both MVPA and sedentary time [34, 37, 45, 48, 59]. The remaining 15 studies examined activity patterns without adjustment for total volume of sedentary time and/or physical activity [35, 39, 43, 44, 46, 47, 49–57]. Consequently, the decision was made to synthesize fully-adjusted models where possible, otherwise unadjusted models were reported. This maximised the opportunity to examine associations between activity patterns with cardio-metabolic health, regardless of the total volume of physical activity and sedentary time.

There was substantial heterogeneity in the definition of activity patterns, variables adjusted for, and effect estimates of cardio-metabolic risk factors (see S2 and S3 Tables); therefore, a meta-analysis was considered inappropriate for this review [61]. Studies reporting observational associations (i.e. cross-sectional and longitudinal studies) [14, 33, 34, 36–47, 49, 51, 53, 55–60] were reported separately from those examining acute effects (i.e. experimental studies) [35, 48, 50, 52, 54]. The results from observational studies are shown in Table 1 and S4–S9 Tables, with each reference number representing one association examined in the corresponding article. Studies reported significantly beneficial (B), significantly detrimental (D), or no significant evidence of an association (NS) between a specific activity pattern (exposure; e.g., >5-min bout of MVPA) and a cardio-metabolic health risk factor (outcome; e.g., BMI). In order to systematically synthesise the observational findings, previously used strategies were considered [62–65]. Consistent with previous systematic reviews [18, 63, 66, 67], only ‘frequently examined associations’ [63] (i.e., if the specific association was investigated at least four times) were discussed. The bold numbers in the right hand columns of Table 1 and S4–S9 Tables represent that specific activity patterns which were examined at least four times. As some studies reported multiple subgroups, only the frequently (≥4 times) investigated associations across multiple studies (i.e., as opposed to within one study) were discussed in the corresponding paragraphs [63]. Given the diversity of outcomes reported, specific outcomes (e.g. body fat, BMI) were grouped under broader outcomes (e.g., adiposity) and discussed. All reported significance levels for the associations were set at p<0.05.

**Table 1:** Studies reporting beneficial, non-significant and detrimental associations of activity patterns with adiposity risk factors.

***Very Hard Physical Activity patterns***						
**Frequency of bouts**	**Beneficial (B)**	**Non-significant (NS)**	**Detrimental (D)**	**B**	**NS**	**D**
**≤15 s**	Body fat [56]			1	0	0
**≥4 s**		Waist [55]^A^		0	1	0
**15–30 s**	Body fat [56]			1	0	0
**30 s—1 min**		Body fat [56]		0	1	0
**1–3 min**		Body fat [56]		0	1	0
**3–10 min**		Body fat [56]		0	1	0
**Time spent in bouts**	**Beneficial (B)**	**Non-significant (NS)**	**Detrimental (D)**	**B**	**NS**	**D**
**≥4 s**		Waist [55]^A^		0	1	0
**Intensity of bouts**	**Beneficial (B)**	**Non-significant (NS)**	**Detrimental (D)**	**B**	**NS**	**D**
**≥4 s**	Waist [55]^A^			1	0	0
***Vigorous Physical Activity patterns***						
**Frequency of bouts**	**Beneficial (B)**	**Non-significant (NS)**	**Detrimental (D)**	**B**	**NS**	**D**
**≤15 s**	Body fat [56]			1	0	0
**≥4 s**	Waist [55]^A^			1	0	0
**15–30 s**	Body fat [56]			1	0	0
**30 s—1 min**	Body fat [56]			1	0	0
**1–3 min**		Body fat [56]		0	1	0
**3–10 min**		Body fat [56]		0	1	0
**Time spent in bouts**	**Beneficial (B)**	**Non-significant (NS)**	**Detrimental (D)**	**B**	**NS**	**D**
**<1 min**		BMI [39]		0	1	0
**≥4 s**		Waist [55]^A^		0	1	0
**1–2 min**		BMI [39]		0	1	0
**≥2 min**	BMI [39]			1	0	0
**Intensity of bouts**	**Beneficial (B)**	**Non-significant (NS)**	**Detrimental (D)**	**B**	**NS**	**D**
**≥4 s**		Waist [55]^A^		0	1	0
***Moderate-to-Vigorous Physical Activity patterns***						
**Frequency of bouts**	**Beneficial (B)**	**Non-significant (NS)**	**Detrimental (D)**	**B**	**NS**	**D**
**≥5 min**		BMI [51]		1	1	0
**Time spent in bouts**	**Beneficial (B)**	**Non-significant (NS)**	**Detrimental (D)**	**B**	**NS**	**D**
**1–4 min**	BMI [46]	Waist [44]^B^		1	1	0
**1–9 min**	Waist [44]^B^			1	0	0
**5–9 min**	BMI [46]			1	0	0
**≥5 min**	BMI [46]; Waist [44]^B^			2	0	0
**≥10 min**	BMI [46]; Waist [44]^B^			2	0	0
**Pattern types**	BMI ‘Most’ vs. ‘Sporadic’ [58]^C^; Waist ‘Most’ vs. ‘Sporadic’, ‘Medium’ vs. ‘Sporadic’ [58]^C^	BMI ‘Most’ vs. ‘Medium’, ‘Medium’ vs. ‘Sporadic’ [58]^C^; Waist ‘Most’ vs. ‘Medium’ [58]^C^		**3**	**3**	**0**
***Moderate Physical Activity patterns***						
**Frequency of bouts**	**Beneficial (B)**	**Non-significant (NS)**	**Detrimental (D)**	**B**	**NS**	**D**
**≥4 s**	Waist [55]^A^			1	0	0
**≥5 min**	Waist [55]^A^			1	0	0
**Time spent in bouts**	**Beneficial (B)**	**Non-significant (NS)**	**Detrimental (D)**	**B**	**NS**	**D**
**≥4 s**	Waist [55]^A^			1	0	0
**≥5 min**	Waist [55]^A^			1	0	0
**Intensity of bouts**	**Beneficial (B)**	**Non-significant (NS)**	**Detrimental (D)**	**B**	**NS**	**D**
**≥4 s**		Waist [55]^A^		0	1	0
**≥5 min**	Waist [55]^A^			1	0	0
***Light Physical Activity patterns***						
**Frequency of bouts**	**Beneficial (B)**	**Non-significant (NS)**	**Detrimental (D)**	**B**	**NS**	**D**
**≤15 s**		Body fat [56]		0	1	0
**≥4 s**		Waist [55]^A^		0	1	0
**15–30 s**	Body fat [56]			1	0	0
**30 s—1 min**	Body fat [56]			1	0	0
**1–3 min**	Body fat [56]			1	0	0
**3–10 min**		Body fat [56]		0	1	0
**≥5 min**		Waist [55]^A^		0	1	0
**≥10 min**		Body fat [56]		0	1	0
**Time spent in bouts**	**Beneficial (B)**	**Non-significant (NS)**	**Detrimental (D)**	**B**	**NS**	**D**
**≥4 s**		Waist [55]^A^		0	1	0
**≥5 min**		Waist [55]^A^		0	1	0
**Intensity of bouts**	**Beneficial (B)**	**Non-significant (NS)**	**Detrimental (D)**	**B**	**NS**	**D**
**≥4 s**	Waist [55]^A^			1	0	0
**≥5 min**	Waist [55]^A^			1	0	0
***Sedentary patterns***						
**Frequency of bouts/breaks**	**Beneficial (B)**	**Non-significant (NS)**	**Detrimental (D)**	**B**	**NS**	**D**
**<30 min**	BMI [60]^E^	BMI [60]^D^		1	1	0
**1–4 min**	BMI [59]^D^, [59]^E^	Waist [59]^D^, [59]^E^		**2**	**2**	**0**
**5–9 min**	Waist [59]^E^	BMI [59]^D^, [59]^E^; Waist [59]^D^		**1**	**3**	**0**
**10–14 min**		BMI [59]^E^; Waist [59]^D^, [59]^E^	BMI [59]^D^	**0**	**3**	**1**
**15–29 min**		BMI [59]^D^, [59]^E^; Waist [59]^D^, [59]^E^		**0**	**4**	**0**
**≥30 min**		BMI [60]^D^, [60]^E^, [59] ^D^, [59]^E^; Waist [59] ^D^, [59]^E^		**0**	**6**	**0**
**Breaks**	BMI [40], [60]^D^, [59]^D^, [59]^E^; Body fat [57]^2/8^; Waist [38]^1/18^	BMI [37], [37]^F^, [37]^G^, [38]^18/18^, [60]^E^; Body fat [57]^6/8^; Waist [38]^17/18^, [53], [59]^D^, [59]^E^, [34]		**7**	**49**	**0**
**Time spent in bouts/breaks**	**Beneficial (B)**	**Non-significant (NS)**	**Detrimental (D)**	**B**	**NS**	**D**
**<30 min**		BMI [40]^H^; Skinfolds [40]^H^		0	2	0
**1–4 min**		BMI [37], [37]^F^	BMI [37]^G^	0	2	1
**≥5 min**		BMI [33]	Waist [33]	0	1	1
**5–9 min**		BMI [37]	BMI [37]^F^, [37]^G^	0	1	2
**≥10 min**			BMI [33]; Waist [33]	0	0	2
**10–19 min**		BMI [37], [37]^F^, [37]^G^		0	3	0
**≥20 min**		BMI [33], [38]^18/18^; Waist [33], [34], [38]^18/18^		**0**	**39**	**0**
**20–29 min**		BMI [37], [37]^F^, [37]^G^		0	3	0
**≥30 min**		BMI [37], [37]^F^, [37]^G^, [40]^H^; Skinfolds [40]^H^; Waist [33], [36]	BMI [33]	**0**	**7**	**1**
**≥40 min**		BMI [38]^18/18^; Waist [38]^17/18^	Waist [38]^1/18^	**0**	**35**	**1**
**≥60 min**		BMI [38]^18/18^; Waist [38]^18/18^		**0**	**36**	**0**
**≥80 min**		BMI [38]^17/18^; Waist [38]^17/18^	BMI [38]^1/18^; Waist [38]^1/18^	**0**	**34**	**2**
**≥100 min**		BMI [38]^18/18^, Waist [38]^18/18^		**0**	**36**	**0**
**≥120 min**		BMI [38]^18/18^; Waist [38]^18/18^		**0**	**36**	**0**
**Breaks**		Skinfolds [40]^H^; Waist [53], [36]^H^		0	3	0
**Intensity of breaks**	**Beneficial (B)**	**Non-significant (NS)**	**Detrimental (D)**	**B**	**NS**	**D**
**Breaks**		Waist [53]		0	1	0
**Duration of bouts/breaks**	**Beneficial (B)**	**Non-significant (NS)**	**Detrimental (D)**	**B**	**NS**	**D**
**≥20 min**		Waist [34]		0	1	0
**Usual bout length**		BMI [41], [42], [42]^I^		0	3	0
**Breaks**		Waist [34]		0	1	0
**Sedentary fragmentation**	**Beneficial (B)**	**Non-significant (NS)**	**Detrimental (D)**	**B**	**NS**	**D**
**Number of sedentary bouts/total hours sedentary**		BMI [45], [45]^I^; Body fat [45], [45]^I^	Body fat [45]^I^; Body fat [45]^I^	**0**	**4**	**2**
***Combined patterns***						
**Activity Fragmentation**	**Beneficial (B)**	**Non-significant (NS)**	**Detrimental (D)**	**B**	**NS**	**D**
**Intradaily Variability (indication of changes between high and low activity)**	BMI [43]; Waist [43]; Waist-to-height ratio [43]; Body fat (5 measures) [43]; Skinfolds [43]	Weight [43]		**9**	**1**	**0**

Abbreviations; *B* Beneficial, *D* Detrimental, *NS* Non significant, *min* Minutes, *s* Seconds, *BMI* Body Mass Index, *Waist* Waist circumference.

^A^ Only minimum borders for intensities were used to classify ≥light, ≥moderate, ≥vigorous and ≥very hard physical activity bouts;

^B^ The sample was divided in four quartiles and the odds ratio of beneficial health factors were presented. When the odds consistently increased/decreased in all quartiles, we assumed that the associations were significantly beneficial/detrimental;

^C^ Latent profile analyses divided sample in ‘Sporadic’, ‘Medium’, and ‘Most bouts’ pattern types. The percentage of MVPA accumulated in sporadic bouts (<5-min) was progressively lower, while the percentage MVPA in both short (5-10-min) and medium-to-long bouts (≥10-min) was progressively higher moving from ‘Sporadic’, to ‘Medium’, and ‘Most bouts’. The underlined pattern type was found beneficial compared to the alternative pattern type;

^D^ Boys;

^E^ Girls;

^F^ Weekdays;

^G^ Weekend days;

^H^ Percentage of time spent in intensity/percentage of sedentary time spent in breaks;

^I^ Longitudinal results.

^X/18^ Colley and colleagues reported associations between activity patterns and cardio-metabolic risk factors from 6 different subgroups (i.e. boys vs. girls in three different age groups; 6–10, 11–14, and 15–19 years) for 3 different time periods (e.g., after-school) [38]. X represents the number of associations categorised as beneficial, non-significant, or detrimental out of the total 18 associations tested.

^X/8^ Kwon and colleagues reported associations between activity patterns and cardio-metabolic risk factors from 8 different subgroups (i.e. boys vs. girls in four different age groups; 8, 11, 13 and 15). X represents the number of associations categorised as beneficial, non-significant, or detrimental out of the total 8 associations tested.

The bold numbers in the right hand columns tables represent that specific activity patterns which were examined at least four times.

## Results

### Review statistics

**Fig 1:**
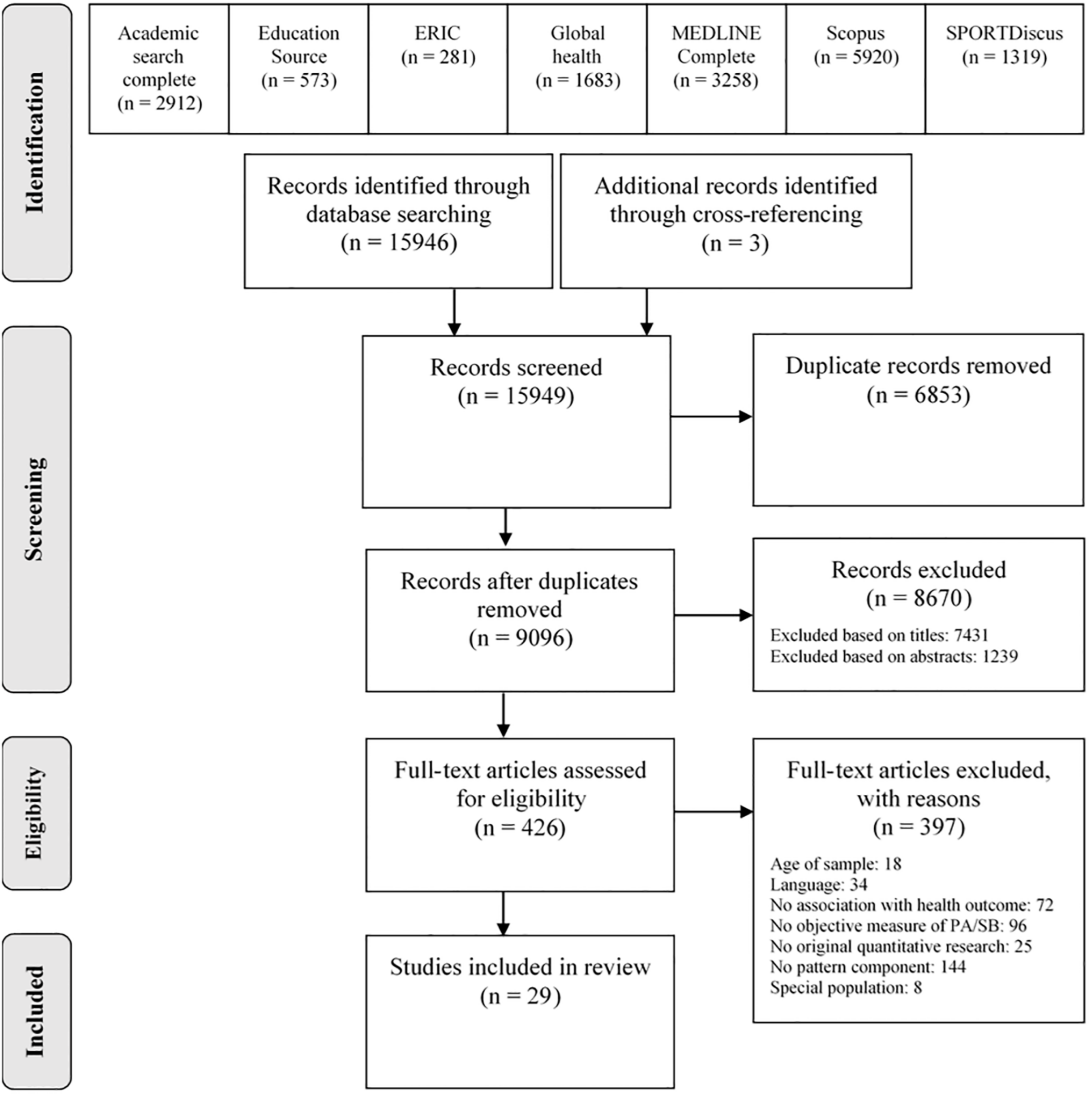
Flow chart of the systematic literature search. From: PRISMA Group [29].

Extracted data were analysed in October-November 2017. A flowchart of the systematic literature search following PRISMA guidelines [29] is presented in Fig 1.

Detailed characteristics of the 29 included articles are presented in S3 Table. Sixteen studies were conducted in North America [35–39, 41, 44, 46–49, 51, 52, 57–59], eight in Europe [33, 34, 40, 43, 45, 55, 56, 60], and five in Oceania [14, 42, 50, 53, 54]. Sample sizes ranged from nine to 2754 participants. Thirteen studies focused on children aged 5-≤12 years only [14, 35, 37, 39, 44, 47, 51–53, 55, 56, 58, 59], six papers focused only on adolescents aged ≥12–18 years [40–43, 49, 50], and ten papers focused on both age groups [33, 34, 36, 38, 45, 46, 48, 54, 57, 60]. Four papers used data drawn from the same sample (U.S. National Health and Nutrition Examination Survey; NHANES 2003–06) [36, 41, 44, 46]. All studies were published from 2009 onwards, with 13/29 (45%) [14, 33–35, 41–43, 45, 50, 52, 54, 58, 60] papers published from 2015 onwards.

S3 Table shows all significant findings reported in the included studies. Ten studies examined physical activity patterns (including VHPA, Vigorous Physical Activity [VPA], MVPA, Moderate Physical Activity [MPA] and Light Physical Activity [LPA]) [39, 44, 46, 47, 49, 51, 55, 56, 58], while 19 studies examined sedentary patterns [14, 33–38, 40–42, 45, 48, 50, 52–54, 57, 59, 60]. Though some studies acknowledged other intensities by adjusting for total volumes of physical activity or sedentary time, only one study specifically examined patterns across the activity spectrum (i.e., low and high intensities) [43]. One study adjusted for total volume of LPA, when examining sedentary bouts and breaks [59]. Detailed information on the adjustments used in each study, including total volumes, are shown in S3 Table.

The patterns assessed mainly consisted of the number of physical activity/sedentary bouts/breaks of a certain duration (e.g., the number of ≥5-min MVPA bouts), the total time spent in physical activity/sedentary bouts of a certain duration (e.g., minutes spent in ≥5-min MVPA bouts), or the intensity of activity/sedentary bouts (e.g., how high was the intensity of ≥5-min bouts). The fragmentation of physical activity and sedentary behaviour and distinct classes of physical activity patterns were also assessed, albeit less frequently. In observational studies, definitions of activity patterns varied substantially in terms of bout lengths (e.g., ≥4-s to ≥20-min bouts for physical activity; ≥1-min to ≥2-h bouts for sedentary behaviour). Of the studies investigating sedentary patterns, ten included breaks (i.e., a non-sedentary bout in between two sedentary bouts [3]) in their assessment of sedentary patterns [14, 34, 36–38, 40, 53, 57, 59, 60]. In the experimental studies [35, 48, 50, 52, 54], all used cross-over designs that included at least two conditions, of which one was uninterrupted sitting. The uninterrupted sitting conditions ranged from three to eight hours. The applied intensities for active interruptions were based on percentages of the ventilatory threshold [35, 52] and VO_2_-peak [48], as well as with preselected activity intensities (measured using cut-points; [54]) or standardised exercises [50].

Thirty-seven different cardio-metabolic risk factors were included in the review and are presented in S2 and S3 Tables. For the purpose of this review, these cardio-metabolic risk factors were classified into the following health outcomes; adiposity, blood lipids, inflammatory and endothelial function biomarkers, glucose metabolism markers, vascular health, fitness, cardio-metabolic summary scores and cortisol.

### Risk of bias assessment

Three (10%) studies had the highest possible rating (i.e., low ROB) on the selection bias components, 11 (38%) adjusted for the desired confounders, ten (34%) used valid measures of activity patterns and cardio-metabolic risk factors, and 13 (45%) had minimal withdrawals and dropouts. Consequently, five articles (17%) were classified as ‘low ROB’ [14, 37, 40, 45, 60], 15 (52%) as ‘medium ROB’ [33–36, 38, 39, 41–44, 46, 48, 51, 53, 56], and nine (31%) as ‘high ROB’ [47, 49, 50, 52, 54, 55, 57–59]. Total ROB scores for each study are presented in S3 Table.

### Observational associations between activity patterns and cardio-metabolic risk factors

#### Adiposity

Twenty-one studies examined cross-sectional associations [33, 34, 36–41, 43, 44, 46, 51, 53, 55–60], one examined longitudinal associations [45] and one examined both cross-sectional and longitudinal associations [42] between activity patterns and adiposity (see Table 1). Outcomes included BMI (including z-scores/percentiles), body fat/fat mass, waist circumference, and skinfolds.

Due to the diversity of bout definitions, no specific combinations between physical activity bouts (including VHPA, VPA, MPA and LPA activity bouts) of a certain duration and an adiposity outcome were frequently investigated (≥4 times) across studies [39, 44, 46, 51, 55, 56, 58]. However, visual inspection of Table 1 shows that there was evidence for significant beneficial associations between physical activity bouts and adiposity (54% of tested associations), regardless of duration and intensity, compared to no significant evidence on the remaining tested associations (46%). No detrimental associations were found for any physical activity patterns.

There was more consistency in the definition of sedentary time patterns in the adiposity studies [33, 34, 36–38, 40–42, 45, 53, 57, 59, 60] (see Table 1). Most of these associations were tested for multiple subsamples within one study (either [38] or [48]). Nevertheless, associations between the number of ≥30-min sedentary bouts [59, 60], and the time spent in ≥20-min [33, 34, 38] and ≥30-min sedentary bouts and adiposity were investigated across multiple studies [33, 36, 37, 40]. No consistent evidence of an association was found. Whilst many studies investigated the associations between sedentary breaks (i.e., frequency) [34, 37, 38, 40, 53, 57, 59, 60] and adiposity, and some evidence of beneficial associations was found, there was no compelling evidence of an association. From 56 associations tested over eight studies [34, 37, 38, 40, 53, 57, 59, 60], seven significantly beneficial associations between sedentary breaks and adiposity were found, compared to 49 tested associations showing null associations. Most studies found mixed evidence across different samples and/or time points [38, 57, 59, 60].

No differences in findings were observed between studies focusing on children [37, 39, 44, 51, 53, 55, 56, 58, 59], adolescents [40–43], or both [33, 34, 36, 38, 45, 46, 57, 60]. In addition, there was little evidence that the findings were influenced by sex (i.e., studies focusing on either sex or separating their sample by sex did not find consistent evidence either) or by study design (i.e., cross-sectional studies and longitudinal studies both showed contrasting results within and across studies).

#### Blood lipids

Eight cross-sectional studies examined associations between activity patterns and blood lipids [33, 34, 36, 38, 43, 44, 58, 59] (see S4 Table). Risk factors included total cholesterol, HDL cholesterol, LDL cholesterol, non-HDL cholesterol, and triglycerides. Only associations between blood lipids and the time spent in ≥20-min [33, 38] and ≥30-min sedentary bouts [33, 36], as well as the number of sedentary breaks [34, 38, 59], were assessed frequently across studies. However, there was no consistent evidence of an association [33, 34, 36, 38, 59]. No studies examined associations between patterns of LPA, MPA, or VPA and blood lipids were examined.

#### Inflammatory and endothelial function biomarkers

Five studies [14, 36, 44, 55, 59] examined associations between activity patterns and inflammatory and endothelial biomarkers (see S5 Table). Inflammatory and endothelial biomarkers included were acetylcholine, C-reactive protein, adiponectin, BDNF, Interleukin-2, -6, -8 and -10, PAI-1, Resistin, sE-selectin, sICAM-1, sVCAM-1, and TNF-α. While two studies examined associations between physical activity patterns (MVPA and LPA bouts only) and two biomarkers (i.e. C-reactive protein and Acetylcholine) [44, 55], no associations were tested more than four times. Three studies investigated associations between sedentary patterns and blood biomarkers [14, 36, 59]. Only sedentary breaks and their potential associations with inflammatory and endothelial biomarkers were studied four or more times across studies. However, these did not show evidence of an association.

#### Glucose metabolism biomarkers

Seven studies [14, 33, 34, 43, 49, 58, 59] examined associations between activity patterns and glucose metabolism markers (see S6 Table). Glucose metabolism biomarkers included were C-peptide, glucose, insulin, intravenous glucose intolerance (K_g_), and HOMA-IR. Two studies investigated physical activity patterns [49, 58] and included MVPA [58] and MPA [49] accumulation. No consistent evidence of a significant association was found. Only the associations between breaks and glucose metabolism markers were examined over multiple studies [14, 34, 59], though no significant associations were found.

#### Vascular health

Six studies investigated associations between activity patterns and vascular health risk factors [34, 36, 38, 44, 47, 58] (see S7 Table). Risk factors included diastolic and systolic blood pressure, and large and small artery compliance. Three studies examined associations between activity bouts and vascular health risk factors [44, 47, 58]. No associations were examined more than four times across studies. Three studies investigated associations between sedentary patterns and vascular health risk factors [34, 36, 38]. Seven patterns were investigated more than four times, however, only the association between the number of breaks in sedentary time and vascular health was assessed in multiple studies [34, 38]. No evidence for an association was found.

#### Fitness

Five studies examined associations between physical activity patterns and fitness [34, 43, 55, 58, 60] (see S8 Table). Fitness outcomes included cardiovascular endurance, a fitness composite score and VO_2_-peak. Included patterns encompassed a broad range of intensities (i.e. VHPA, VPA, MVPA, MPA, LPA, and sedentary time) and combined intensities, yet none were frequently examined across studies.

#### Cardio-metabolic summary scores

Seven studies investigated associations between activity patterns and cardio-metabolic risk summary scores [33, 34, 36, 41, 43, 44, 59] (see S9 Table). Nine different methods of calculating cardio-metabolic risk scores were used and are briefly described in the footnotes of S9 Table. Only the associations between breaks and cardio-metabolic summary scores were examined over multiple studies [59, 34], though no consistent significant association was found.

### Experimental studies

Five experimental studies were included in this review [35, 48, 50, 52, 54]. The acute effects of (un-)interrupted prolonged sitting were investigated by applying intensities based on the ventilatory threshold [35, 52] and VO_2_-peak [48], or activity intensity cut-points [54] and preselected standardised exercises [50] for their sit and walk/exercise protocols. The uninterrupted sitting conditions were of three [35, 52], six [50, 54] and eight [48] hours duration.

Two experimental studies used a sitting protocol and investigated children aged between and 7–11, however, found contrasting results [35, 52]. Belcher and colleagues [35] tested whether three hours of interrupting sitting with moderate-intensity walking bouts every 30 min improved glucose tolerance and found that interrupted sitting resulted in significantly lower insulin, C-peptide and glucose [35]. McManus and colleagues utilised a protocol with moderate-intensity cycling bouts to interrupt sitting every hour. No significant differences between conditions were observed [52].

Fletcher and colleagues [50] focused on adolescents and compared uninterrupted sitting with 2-min activity breaks involving LPA body-weight resistance activities every 18 min. Compared to uninterrupted sitting, the breaks condition elicited a lower postprandial glucose area under the curve response after the first and second standard-energy meal, however, not for the entire trial period or for total area under the curve [50]. Similarly, Ross and colleagues [54] found no differences in triglyceride concentration between six hours uninterrupted sitting and sitting interrupted by short bouts of moderate intensity exercise every 30 min in children and adolescents. Saunders and colleagues [48] examined compared eight hours of uninterrupted sitting with eight hours of sitting interrupted by light intensity walking breaks every 20 min or structured physical activity and found no significant differences in insulin, glucose, triglyceride, HDL cholesterol, and LDL cholesterol in children and adolescents [48].

While similar protocols have been used across experimental studies included in this review [35, 48, 50, 52, 54], the risk factors assessed have been inconsistent. Some evidence suggests that breaking up sitting may influence glucose [35, 50]; few consistent significant results have been found in relation to specific patterns of activity.

### Risk of Bias and associations between activity patterns and cardio-metabolic risk factors

Fifteen studies found evidence of a significant beneficial association between an activity pattern and a cardio-metabolic risk factor [35, 38–40, 43, 44, 46, 49, 50, 55–60]. In contrast, seven studies found evidence of a significant detrimental association between an activity pattern and a cardio-metabolic risk factor [33, 34, 37, 38, 43, 45, 59]. Of these studies (i.e., the 19 studies finding any significant results), six (32%) were of high ROB [49, 50, 55, 57–59], nine (47%) were of medium ROB [33–35, 38, 39, 43, 44, 46, 56], and four (21%) were of low ROB [37, 40, 45, 60]. The remaining ten studies did not finding any significant results. Of these studies, three (30%) were of high ROB [47, 52, 54], six (60%) were of medium ROB [36, 41, 42, 48, 51, 53] and one (10%) was of low ROB [14]. ROB scores for each study are presented in S3 Table.

## Discussion

This systematic review examined the effect of objectively-measured activity patterns across the activity spectrum on cardio-metabolic risk factors in children and adolescents. Based on summative evidence coding used on observational study findings [63], several associations were investigated frequently yet no consistent evidence of beneficial and/or detrimental associations were observed. Whilst stronger results were anticipated for adolescents because of potential greater exposure to unhealthy lifestyle behaviours compared to children, inconsistent evidence was found regardless of whether studies focused on children and/or adolescents. In addition, results did not differ by study design, differing ROB levels, and whether the activity patterns were adjusted for total physical activity and/or sedentary time or not. The five experimental studies also showed inconsistent results. Two of these suggested that interrupting sedentary time could be beneficial for short-term metabolic function [35, 50], but no evidence for associations between interrupting prolonged sitting was found among the remaining studies [48, 52, 54].

The findings from this review are consistent with those from systematic reviews by Cliff and colleagues [18] and Carson and colleagues [15] that focused on the associations between sedentary time and patterns and health and well-being in children and adolescents. Although little detail was provided about the specific patterns of sedentary time examined, both reviews reported that only a few studies had examined patterns of sedentary time. Carson and colleagues [15] noted that sedentary bouts and breaks were not consistently associated with any health outcome. In contrast, a systematic review that focused on total physical activity and physical activity patterns concluded that all patterns (i.e., sporadically and in continuous bouts) provided benefit for health [11]. This contrasts the results of the current review, which found no consistent evidence across studies to support the benefits of either prolonged and/or sporadic physical activity patterns on cardio-metabolic risk factors. These different findings might be due to our decision to use summative evidence coding [63], rather than considering each individual finding separately. Whilst summative coding may provide clarity around which activity patterns have been consistently investigated, many activity patterns did not meet the criteria to be coded as evidence (i.e. ≥4 times). Notably, none of the patterns in the review by Poitras and colleagues [11] would have met the criteria to be included in the synthesis of the current review. The lack of consistent significant associations identified in this review may potentially be explained by the high ROB across the studies. This was particularly due to insufficient statistical adjustments for confounders and inadequate methods for dealing with ‘withdrawals and drop-outs’. Only five studies were assessed as ‘low ROB’ [14, 37, 40, 45, 60]. However, it is important to note that the findings within this systematic review were consistent across ROB categories. In other words, the observed associations did not differ per ROB category (i.e., low, medium, high). Previous reviews focusing on activity and health in this age group have also noted concerns about high ROB [11, 15, 18]. For example, Cliff and colleagues’ [18] review, which focused on sedentary behaviours and health in children and adolescents, found that less than 50% of their studies were of ‘low ROB’.

The majority of included studies were cross-sectional (76%); there was a dearth of longitudinal studies. Therefore, it is not possible to make appropriate conclusions on cause and effect. Interestingly, the two studies with longitudinal designs [42, 45] found contrasting results. Future additional longitudinal studies would provide more insights into potential causal relationships for the effects of activity patterns on health.

### Strengths and limitations

This review is the first to examine associations between specific activity patterns across the entire activity spectrum and a wide range of cardio-metabolic risk factors in children and adolescents. To date, most reviews that have examined activity patterns have not considered which specific activity patterns of activity may be important for health as their primary focus was on total volumes of physical activity and/or sedentary behaviour. Typically, these reviews do not distinguish between different patterns (e.g. frequency, duration, and intensity of bouts) (e.g., [15, 18]), despite this information having the potential to inform future interventions and public health guidance on how to accumulate physical activity and sedentary behaviour (e.g., bouts of particular durations) to benefit health.

There are several limitations which should be taken into account, such as the use of summative evidence coding approach [63] and that some papers (e.g., [38]) included multiple subgroups and/or time points and consequently dominated the evidence database. This may have led to erroneous interpretation of potential beneficial or detrimental associations. However, summative evidence coding [63] is a commonly used approach within this field of research, and did allow more systematic comparisons across studies than a narrative review. The reason for not undertaking a narrative synthesis is that such approaches typically describe and discuss the state of the literature, providing a narrative interpretation and critique of a broader area [68]. Given the number of studies included, this review focused on summarising data [68] from the different studies to highlight how activity patterns are associated with health, and to identify existing gaps. Ultimately, this review highlights the variability defining activity patterns and limitations in evidence, and is the first step towards a more standardised assessment. Nevertheless, following data reporting, results were checked to explore if the results would have differed if we would had only reported one association per study (regardless of the total number of associations reported within a study), but this did not make any difference to the conclusions.

There are also a number of methodological issues to consider that may have influenced the ability to detect associations. Firstly, there were differences in the confounders across studies. Some studies adjusted for total physical activity and/or sedentary time and some did not. Fully-adjusted models were included to examine activity patterns, regardless of total time spent in different intensities. In addition, the chosen intensity thresholds and corresponding cut-points, and epoch lengths (i.e., 2 s to 60 s) varied amongst included studies. Children and adolescents have sporadic activity patterns, with the majority of high intensity activities lasting less than 10 s [24, 69]. Shorter epoch lengths have been shown to minimise errors in measuring these sporadic behaviours in children [70, 71]. Overall, the variation in confounder adjustment, chosen intensity thresholds and corresponding cut-points, and epoch lengths, may have contributed to the lack of consistent associations between activity patterns and risk outcomes observed.

Despite the aforementioned issues, this review identified a number of gaps in current knowledge. Firstly, most studies (21/29; 72%) focused on markers of adiposity (e.g., BMI, waist circumference). Fewer studies have investigated associations between activity patterns and blood biomarkers such as lipids (28%), inflammatory and endothelial biomarkers (17%), or glucose metabolism biomarkers (24%). This is consistent with a review focusing on relationships of combinations of physical activity, sedentary behaviour and sleep with health indicators, also in school-aged children and adolescents, which found that other health factors than adiposity have been under-researched to date [72].

The current review also found that most research has focused on either sedentary bouts/breaks or MVPA bouts, with only two of the 29 studies examining LPA patterns [55, 56]. Whilst some studies acknowledge the importance of other intensities by adjusting for them, none have examined multiple intensity patterns together (e.g., using cluster analyses, compositional analyses [73, 74]). Young people do not engage in activity intensities in isolation and there are many ways to accumulate time through different combinations of sedentary behaviours and physical activity [73, 74]. Specifically, when a child is engaging in one activity intensity, they cannot be engaging in another activity intensity. It is these combinations of activity patterns (e.g., how often and with what intensity should you break up sitting) that have been understudied [73, 74]. While there is already some recognition of conceptualising sedentary time and physical activity (including all intensities such as LPA and MPA) together as part of a continuum in total volume research [72, 74], most studies have only investigated independent effects of patterns for a single intensity. Consequently, it is not known whether differential associations are observed between combined activity patterns and cardio-metabolic risk factors.

### Future directions

Whilst this review highlights that there is emerging interest in examining associations between activity patterns and cardio-metabolic risk factors in children and adolescents (100% of studies were published from 2009 onwards, and 45% from 2015 onwards), few studies have consistently examined the same activity patterns with cardio-metabolic risk factors. This made it impossible to recommend how children and adolescents should accumulate their activity to benefit their cardio-metabolic health. To aid comparability between studies, it would be beneficial for future research to consider standardised pattern definitions and assessments of activity patterns (e.g., accepted methodology and terminology for assessing sporadic and prolonged activity patterns). This would enable studies to replicate these assessments in different populations, thus building the evidence base in relation to whether activity patterns impact cardio-metabolic health outcomes in children and adolescents. More evidence is also needed for cardio-metabolic risk factors other than adiposity, such as blood lipids. This research should include the full activity spectrum from sedentary to vigorous, and the composition of different activity patterns (e.g., combined activity patterns of bouts and breaks of multiple intensities). As it is possible that there will be differing association with cardio-metabolic health depending on the combination of activity patterns across the activity spectrum [73, 74], it is important that future research also examines associations of these combinations and cardio-metabolic health in children and adolescents. This will contribute to the understanding of activity patterns in children and adolescents, provide knowledge as to whether existing physical activity and sedentary behaviour guidelines need to be refined to include recommendations relating to specific activity patterns, and enhance the development of targeted interventions to benefit health outcomes.

## Conclusion

In summary, this review found limited consistent evidence of associations between activity patterns across the activity spectrum and cardio-metabolic health in children and adolescents. While this review found little evidence of associations between activity patterns and cardio-metabolic risk factors, it is premature to conclude that activity patterns do not affect cardio-metabolic risk. Substantial variety in pattern definitions made comparisons between studies difficult, therefore a standardised assessment of activity patterns is needed to progress this field of research. Further evidence, including more longitudinal and experimental data covering a range of cardio-metabolic risk factors, is needed to better understand the health impact of children’s and adolescents’ activity patterns. All separate intensities and combined intensities should be considered in future work aimed at understanding the activity patterns of children and adolescents.

## Supporting information

S1 Table Systematic search strategy: Associations between activity patterns and cardio-metabolic risk factors in children and adolescents: A systematic review (PICO principle [30]). (PDF)

S2 Table References grouped per activity pattern. (PDF)

S3 Table Overview of characteristics of included studies (PICO principle [30]). (PDF)

S4 Table Studies reporting beneficial, non-significant and detrimental associations of activity patterns with blood lipids. (PDF)

S5 Table Studies reporting beneficial, non-significant and detrimental associations of activity patterns with inflammatory and endothelial blood biomarkers. (PDF)

S6 Table Studies reporting beneficial, non-significant and detrimental associations of activity patterns with glucose metabolism biomarkers. (PDF)

S7 Table Studies reporting beneficial, non-significant and detrimental associations of activity patterns with vascular health. (PDF)

S8 Table Studies reporting beneficial, non-significant and detrimental associations of activity patterns with fitness. (PDF)

S9 Table Studies reporting beneficial, non-significant and detrimental associations of activity patterns with cardio-metabolic summary scores. (PDF)

S10 Table PRISMA checklist [29]. (PDF)

S1 Text Risk of bias assessment tool (based on items of the ‘EPHPP Quality Assessment Tool for Quantitative Studies’ [31]). (PDF)
