# Supplementary material for: Associations between activity patterns and cardio-metabolic risk factors in children and adolescents: A systematic review
Source: PLoS One. 2018 Aug 16;13(8):e0201947. doi: 10.1371/journal.pone.0201947 (PMC6095515; doi:10.1371/journal.pone.0201947)
Supplement: S1 Table — (PDF) [file pone.0201947.s001.pdf]

S1 Table. Systematic search strategy: Associations between activity patterns and cardio-metabolic risk factors in children and adolescents: A systematic review (PICO principle [30]).

**P**opulation	**Child***	OR Kid* OR Youth OR Adolescen* OR Teen* OR Student* OR Juvenile* OR "Young person" OR “Young people” OR “Primary school*” OR “Secondary school*” OR “High school*” OR “Elementary school*”
**I**ntervention/Exposure	**“Physical* activ*”**	OR Exercis* OR Sedentar* OR Sitting OR Standing OR “Aerobic activ*” OR “Energy expenditure”
	**Objective***	OR Observ* OR Acceleromet* OR Inclinometer OR Pedometer OR SenseWear OR CSA OR MTI OR ActiGraph OR ActivPal OR GENEactiv OR Minimitter OR “Mini-Mitter” OR IDEEA OR Caltrac OR Actiheart
	**Pattern***	OR Bout* OR Break* OR Period* OR “Within-Day” OR "Between-Day" OR Segments OR Transitions or “Time Use”
	**Guideline***	OR Recommendation*
**C**omparator	**NA**	NA
**O**utcome	**Health**	OR "Risk Factor*"
	**Cardiometabolic**	OR Insulin OR Glucose OR Metabolic OR "Syndrome X" OR "Deadly Quartet" OR Plurimetabolic OR Blood OR Cholesterol* OR Hypercholesterol* OR “Blood pressure” OR Hypertension OR Triglycerid* OR Lipid* OR Dyslipid* OR Inflamm* OR Cardiovascular OR Diabetes
	**Obes***	OR Adiposity OR Overweight OR “Body fat” OR “Body composition” OR “Waist circumference” OR "Skin fold*" OR “Body mass index” OR “Hip circumference” OR BMI OR “Bio-impedance analysis” OR “Dual-energy x-ray absorptiometry” OR DXA OR DEXA
	**Fitness**	OR Cardiorespiratory OR Musc* OR Strength OR Endurance OR Conditioning

**NOT Statement**	Animal* OR rat* OR mouse OR mice (TITLE-ABS-KEY)
**Total search**	((S1 AND S2 AND S3 AND S4) AND (S5 OR S6 OR S7 OR S8 OR S9)) AND NOT TITLE-ABS-KEY (animal* OR rat* OR mouse OR mice))
**Limiters for date, language and human studies**<br>Limiters - Published Date: 1980/01/01 – 2017/10/31<br>Language (Dutch/Flemish, English): Academic search complete, Education Source, Medline Complete.<br>Studies (Human): Global health (Broad cat: Human Sciences).	
**Search Strategy**<br>(TITLE-ABS-KEY ("Physical* activ*" OR exercis* OR sedentar* OR sitting OR standing OR "Aerobic activ*" OR "Energy expenditure") AND TITLE-ABS-KEY (child* OR kid* OR youth OR adolescen* OR teen* OR student* OR juvenile* OR "Young person" OR "Young people" OR "Primary school*" OR "Secondary school*" OR "High school*" OR "Elementary school*") AND TITLE-ABS-KEY (objective* OR observ* OR acceleromet* OR inclinometer OR pedometer OR sensewear OR csa OR mti OR ActiGraph OR activpal OR geneactiv OR minimitter OR "Mini-Mitter" OR ideea OR caltrac OR actiheart) AND TITLE-ABS-KEY (pattern* OR bout* OR break* OR period* OR "Within-Day" OR "Between-Day" OR segments OR transitions OR "Time Use") AND TITLE-ABS-KEY (health OR "Risk Factor*") OR TITLE-ABS-KEY (cardiometabolic OR insulin OR glucose OR metabolic OR "Syndrome X" OR "Deadly Quartet" OR plurimetabolic OR blood OR cholesterol* OR hypercholesterol* OR "Blood pressure" OR hypertension) OR TITLE-ABS-KEY (triglycerid* OR lipid* OR dyslipid* OR inflamm* OR cardiovascular OR diabetes) OR TITLE-ABS-KEY (obes* OR adiposity OR overweight OR "Body fat" OR "Body composition" OR "Waist circumference" OR "Skin fold*" OR "Body mass index" OR "Hip circumference" OR bmi OR "Bio-impedance analysis" OR "Dual-energy x-ray absorptiometry") OR TITLE-ABS-KEY (dxa OR dexa) OR TITLE-ABS-KEY (fitness OR cardiorespiratory OR musc* OR strength OR endurance OR conditioning) OR TITLE-ABS-KEY (guideline* OR recommendation*) AND NOT TITLE-ABS-KEY (animal* OR rat* OR mouse OR mice)) AND PUBYEAR > 1979	

References *(reference numbers correspond with manuscript)*

30. Deeks JJ, Higgins JPT, Altman DG. 5.1.1 Rationale for well-formulated questions. In: Higgins JPT, Green S, editors. Cochrane Handbook for Systematic Reviews of Interventions. 5.1.0 ed. London: The Cochrane Collaboration; 2011.
