# Supplementary material for: Associations between activity patterns and cardio-metabolic risk factors in children and adolescents: A systematic review
Source: PLoS One. 2018 Aug 16;13(8):e0201947. doi: 10.1371/journal.pone.0201947 (PMC6095515; doi:10.1371/journal.pone.0201947)
Supplement: S3 Table — (PDF) [file pone.0201947.s003.pdf]

S3 Table. Overview of characteristics of included studies (PICO principle [30]).

**P I C O:**	**P**opulation					**I**ntervention/Exposure					**O**utcome			
**Reference[A]**	**Design**	**Country**	**N**	**Sex[B]**	**Age (mean ± SD)**	**Device/ Method**	**Epoch (s)**	**Intensity thresholds (METs)**	**Accelerometer cut-points (cpm)[C]**	**Patterns**	**Health outcomes**	**Significant findings**	**Adjustments**	**ROB score**
**Altenburg (2015) [33]**	CS	Europe[D]	647	Mixed	11.6 ± 0.8	AG, At	15/60	N/R	SED <100<br>MVPA ≥3000	SED: Time spent in ≥5, ≥10, ≥20, ≥30-min bouts	BMI, WC, C-peptide, glucose, HDL-C, LDL-C, TC, TG, CMR-score	BMI (D), WC (D), C-peptide (D), CMR-score (D)	Age, sex, MVPA, wear time, country. Fasting blood levels: additionally WC.	Med risk
**Bailey (2017) [34]**	CS	UK	111	Mixed	11.8 ± 1.4	RT3	60	SED <1.5	SED <420<br>LPA 420-1859<br>MVPA ≥1860	SED: Frequency of ≥20-min bouts and breaks & mean duration of ≥20-min bouts and breaks	WC, glucose, TC, HDL-C, TG, SBP, DBP, CMR-score (2x), fitness	TG (D), CMR-score (D)	Sex, ethnicity, SED, MVPA, wear time.	Med risk
**Belcher (2015) [35]**	EXP	USA	28	Mixed	10.2 ± 1.5	% of VT	N/A	N/A	N/A	SED: 180 min uninterrupted vs. breaks every 30 min	C-peptide, glucose, insulin, TG, FFA, cortisol	C-peptide (B), glucose (B), insulin (B)	Baseline serum values, age, sex, BF, randomization order, visit condition, puberty.	Med risk
**Blaes (2011) [56]**	CS	France	187	Boys[E]	9.1 ± 1.3	AG	5	LPA <3<br>MPA 3-6<br>VPA 6-9<br>VHPA >9	LPA <162<br>MPA 163-440<br>VPA 441-790<br>VHPA >790	LPA: Frequency of 5-15, 16-30, 31-60 s, 1-3, 3-10 and >10-min bouts<br>VPA/VHPA: Frequency of 5-15, 16-30, 31-60 s, 1-3, 3-10-min bouts	BF	BF (B)	Age and BF.	Med risk

**Carson (2011) [36]**	CS	USA	2527	Mixed	13 ± 4.5	AG	60	MVPA >4	SED <100 LPA Freedson MVPA Freedson	SED: Time spent in ≥30-min bouts and % of bout time spent in breaks	WC, CRP, non HDL-C, CMR-score, SBP	NS	Age, sex, ethnicity, SES, smoking, BF, fat, saturated fat, dietary cholesterol, sodium, MVPA.	Med risk
**Carson (2014) [37]**	CS	Canada	787	Mixed	11.1 ± 0.7	AG	5	MVPA >4	SED ≤100 LPA, MVPA Freedson	SED: Time spent in 1-4, 5-9, 10-19, 20-29, ≥30-min bouts & frequency of breaks/hr for total week, week day and weekend days.	BMI	BMI (D)	Age, sex, SES. SED patterns: additionally MVPA. SED breaks and MVPA: additionally SED.	Low risk
**Colley (2013) [38]**	CS	Canada	1608	Separate	6-19 (mean ± SD N/R)	Ac	60	SED <1.5	SED ≤100 MVPA ≥1500	SED: Time spent in ≥20, ≥40, ≥60, ≥80, ≥100, ≥120-min bouts & frequency of breaks	BMI, WC, non HDL, DBP, SBP	BMI (D), WC (B), WC (D)	Age, MVPA, wear time.	Med risk
**Dorsey (2011) [39]**	CS	USA	106	Mixed	9.4 ± 0.9	Ac	15	N/R	SED <100 LPA 100-900 MPA 900-2200 VPA ≥2200	VPA: Time spent in <1, 1-2, ≥ 2-min	BMI	BMI (B)	Age, sex, type of day.	Med risk
**Dowd (2014) [40]**	CS	Ireland	195	Girls	15.7 ± 0.9	aP	15	SED <1.5 MVPA ≥3 LPA = 24h – SED – MVPA[F]	MVPA ≥11988	SED: % time in <30, >30-min bouts & frequency of breaks	BMI, skinfolds	BMI (B)	Age, clustering of participants within schools, MVPA.	Low risk
**Fletcher (2017a) [41]**	CS	USA	1797	Mixed	15.1 ± 2.2	AG	60	SED <1.5	SED ≤100	SED: Median bout length	BMI, CMR-score	NS	Age, sex, ethnicity, SES, dietary intake, MVPA.	Med risk
**Fletcher (2017b) [42]**	CS, LONG	Australia	140	Mixed	12.9 ± 0.2	AG	60	SED <1.5	SED ≤100	SED: Median bout length	BMI	NS	Age, sex, SES, puberty, MVPA.	Med risk

**Fletcher (2017c) [50]**	EXP	Australia	13	Mixed	16.4 ± 1.3	Standard exercises	N/A	N/A	N/A	SED: 360 min uninterrupted vs. 2-min activity breaks every 18 min	Glucose	Glucose (B)	-	High risk
**Gabel (2016) [14]**	CS	Australia	164	Mixed	8.7 ± 0.4	AG	15	SED <1.5 MPA 4-6 VPA ≥6	SED ≤100 MVPA Freedson	SED: Time spent in 5-10 and >10-min bouts & frequency of 5-10, >10-min bouts & breaks	Adiponectin, BDNF, CRP, HOMA-IR, IL-2, IL-6, IL-8, IL-10, PAI-1, resistin, sE-selectin, sICAM-1, sVCAM-1, TNF-α	NS	Clustering within schools, sex, WC, MVPA, diet density.	Low risk
**Garaulet (2016) [43]**	CS	Europe[D]	1044	Mixed	14.5 ± 1.2	AG	15	N/R	MVPA >2000	Fragmentation(Frequency of changes between high and low activity)	BMI, WC, waist-to-height ratio, skinfolds (2x), BF (4x), HDL-C, LDL-C, TC, TG, glucose, HOMA-IR, insulin, DBP, SBP, Fitness, CMR-score (2x)	BMI (B), WC (B), waist-to-height ratio (B), BF (B), skinfolds (B), HDL-C (D), Fitness (D), CMR-score (D)	Age, sex, SES.	Med risk
**Harrington (2013) [51]**	CS	USA	55	Mixed	8	AG	2	LPA <3 MPA 3-6	LPA Freedson MPA Freedson VPA Freedson	MVPA: Frequency of 5-min bouts	BMI	NS	-	Med risk
**Holman (2011) [44]**	CS	USA	2754	Mixed	13 ± 4.5	AG	60	MVPA ≥4	MVPA Freedson	MVPA: Time spent in 1-4, ≥5, 1-9, ≥10-min bouts	WC, CRP, non HDL-C, CMR-score, SBP	WC (B), CRP (B), CMR-score (B), SBP (B)	Sex, age, ethnicity, SES, diet (total fat and sodium), smoking status, wear time.	Med risk

**Júdice (2017) [60]**	CS	Portugal	2698	Mixed	13.4 ± 2.3	AG	60	N/R	SED <100<br>LPA 100-2295<br>MVPA ≥2296	SED: Frequency of <30 min, ≥30-min bouts and breaks	BMI, Fitness	BMI (B), Fitness (B)	SED, sex, age, season of data collection.	Low risk
**Kwon (2013) [57]**	CS	USA	554	Separate	8, 11, 13,15	AG	60	N/R	SED <100<br>MVPA ≥2296	SED: Frequency of breaks	BF	BF (B)	Age, body size, physical maturity.	High risk
**Mann (2017) [45]**	CS, LONG	UK	502 (BL)	Mixed	7.5 ± 0.5 (BL)	AG	15	NR	SED <100<br>MVA ≥2400	SED: Frequency of ≥1-min bouts	BMI, BF	BF (D)	SED (within the pattern variable, divided by SED hours), sex, MVPA, season.	Low risk
**Mark (2009) [46]**	CS	USA	2498	Mixed	12.7 ± 2.8	AG	60	MVPA ≥3	MVPA ≥3000	MVPA: Time spent in 1-4, 5-9, ≥10-min bouts	BMI	BMI (B)	Sex, age, ethnicity, SES.	Med risk
**McManus (2015) [52]**	EXP	Canada	9	Girls	9.4 ± 0.78	% of VT	N/A	N/A	N/A	SED: 180 min uninterrupted vs. 10-min breaks every 60 min	Superficial femoral artery parameters (6x)	NS	-	High risk
**Nettlefold (2012) [47]**	CS	Canada	105	Mixed	9.9 ± 0.6	AG	15	SED <1.5<br>LPA 1.5-3<br>MPA 3-6<br>VPA ≥6	SED Freedson<br>LPA Freedson<br>MPA Freedson<br>VPA Freedson	MVPA: Time spent in 0-5, 5-10, 10-20, ≥20-min bouts	LAC, SAC	NS	Body surface area, SBP, BMI, sex.	High risk
**Oliver (2013) [53]**	CS	New Zealand	126	Mixed	5.9 ± N/R	Ac	60	N/R	SED <100<br>MVPA ≥1500	SED: Frequency, average duration and average intensity of breaks	WC	NS	-	Med risk
**Ross (2015) [54]**	EXP	New Zealand	12	Mixed	11.5 ± 1.57	aP	N/R	N/R	N/R	SED: 360 min uninterrupted vs. 4-min MPA breaks every 30 min	TG	NS	-	High risk

**Saunders (2013a) [48]**	EXP	Canada	19	Mixed	12.2 ± 1.1	% of VO_2-peak	N/A	N/A	N/A	SED: 8 hours uninterrupted sitting vs. 2-min activity breaks every 20 min vs. 2-min activity breaks every 18 min + 2x20 min MVPA	Glucose, insulin, HDL-C, LDL-C, TG	NS	Condition, age, sex, BMI, WC, Tanner stage, baseline PA and SED.	Med risk
**Saunders (2013b) [59]**	CS	Canada	522	Separate	9.2 ± N/R	AG	60	SED <1.5	SED <100 LPA 100-2296 MVPA >2296	SED: Frequency of 1-4, 5-9, 10-14, 15-29 and ≥30 min-bouts & breaks	BMI, WC, CRP, glucose, insulin, HDL-C, TG, CMR-score	BMI (B), BMI (D), WC (B), CRP (B), glucose (D), TG (B)	Wear time, age, LPA, MVPA, SED, BMI, puberty, SES.	High risk
**Stone (2009) [55]**	CS	UK	47	Boys	9.4 ± 0.7	AG	2	MPA 4-6 VPA >6	SED <300 LPA 300-3581 MPA 3581-6130 VPA 6130-9630 VHPA >9630	LPA, MPA, VPA, VHPA: Frequency, intensity & time spent in ≥4-s & ≥5-min bouts	WC, ACh peak, Ach AUC, VO_2-peak	WC (B), ACh peak (B), VO_2-peak (B)	-	High risk
**Thomas (2009) [49]**	CS	USA	32	Mixed	16.0 ± 1.6	AG	60	LPA <3 MPA 3-6 VPA 6-9 VHPA ≥9	LPA ≤ 1952 MPA 1953-5724 VPA 5725-9498 VHPA ≥9499	MVPA: Frequency of 5-min bouts of MVPA	K_g	K_g (B)	Race, sex, BF.	High risk
**Willis (2015) [58]**	CS	USA	391	Mixed	7.6 ± 0.6	AG	60	N/R	MVPA Freedson	MVPA: Comparing 3 classes identified based on the composition of <5, 5-10, ≥10-min bouts/day	BMI, WC, glucose, insulin, HDL-C, TC, TG, DBP, SBP, fitness	BMI (B), WC (B)	Age, sex, BMI, MVPA.	High risk

Abbreviations; *CS* Cross-sectional, *EXP* Experimental, *LONG* Longitudinal, *N* sample size, *cpm* count per min, *min* min, *s* Seconds, *AG* ActiGraph, *At* Actitrainer, *Ac* Actical, *aP* activPAL, *VO_2-peak* Maximal oxygen uptake, *VT* Ventilatory Threshold, *PA* physical activity, *SED* sedentary time, *LPA* light physical activity, *MPA* Moderate Physical Activity, *MVPA* Moderate-to-Vigorous physical activity, *VPA* Vigorous Physical Activity, *VHPA* Very High Physical Activity, *N/R* not reported, *N/A* not applicable, *B* Beneficial association reported, D Detrimental association reported, *NS* No significant results, *BMI* Body Mass Index, *BF* Body fat, *WC* Waist circumference, *ACh* Acetylcholine, *AUC* Area Under Curve, *BDNF* Brain-derived neurotrophic factor, *CRP* C-reactive protein, *FFA* Free fattty acids, K_g Intravenous glucose intolerance, *HOMA-IR* Homeostatic Model Assessment (-Insulin Resistance), *IL* interleukin, *PAI* Plasminogen activator inhibitor, *sICAM* Soluble intercellular adhesion molecule, *sVCAM* Soluble Vascular Cell Adhesion Bolecule, *TG* Triglycerides, *TNF* Tumor Necrosis Factor, *HDL-C* High Density Lipoprotein Cholesterol, *LDL-C* Low Density Lipoprotein Cholesterol, *TC* Total cholesterol, *CV* Cardiovascular, LAC Large Artery compliance, *SAC* Small Artery Compliance, *DBP* Diastolic Blood Pressure, *SBP* Systolic Blood Pressure, *BL* Baseline, *Med* Medium, *Freedson* Age-specific cut-points as developed by Freedson and colleagues [75].

[A] Only name of first author mentioned in table; [B] Mixed: Data were analysed for boys and girls together; Separate: Both sexes were tested and reported separately; Boys: Sample only consisted of boys; Girls: Sample only consisted of girls; [C] Cut-off points given in epochs were calculated to counts per minute; [D] Participants included were from multiple European countries; [E] Initial analyses (total volumes) were separately done for boys and girls. Activity pattern analyses were only done for boys, which are the only used results in the current systematic review; [F] LPA was calculated including and excluding standing time using the activPAL posture measures.

References *(reference numbers correspond with manuscript)*

14. Gabel L, Ridgers ND, Della Gatta PA, Arundell L, Cerin E, Robinson S, et al. Associations of sedentary time patterns and TV viewing time with inflammatory and endothelial function biomarkers in children. Pediatr Obes. 2016;11(3):194-201. doi: 10.1111/ijpo.12045. PubMed PMID: 26097139.

30. Deeks JJ, Higgins JPT, Altman DG. 5.1.1 Rationale for well-formulated questions. In: Higgins JPT, Green S, editors. Cochrane Handbook for Systematic Reviews of Interventions. 5.1.0 ed. London: The Cochrane Collaboration; 2011.

33. Altenburg TM, de Niet M, Verloigne M, De Bourdeaudhuij I, Androutsos O, Manios Y, et al. Occurrence and duration of various operational definitions of sedentary bouts and cross-sectional associations with cardiometabolic health indicators: the ENERGY-project. Prev Med. 2015;71:101-6. doi: 10.1016/j.ypmed.2014.12.015. PubMed PMID: 25535676.

34. Bailey DP, Charman SJ, Ploetz T, Savory LA, Kerr CJ. Associations between prolonged sedentary time and breaks in sedentary time with cardiometabolic risk in 10–14-year-old children: The HAPPY study. J Sports Sci. 2017;35(22):2164-71. doi: 10.1080/02640414.2016.1260150.

35. Belcher BR, Berrigan D, Papachristopoulou A, Brady SM, Bernstein SB, Brychta RJ, et al. Effects of interrupting children's sedentary behaviors with activity on metabolic function: A randomized trial. J Clin Endocrinol Metab. 2015;100(10):3735-43. doi: 10.1210/jc.2015-2803. PubMed PMID: 26312582; PubMed Central PMCID: PMCPMC4596047.

36. Carson V, Janssen I. Volume, patterns, and types of sedentary behavior and cardio-metabolic health in children and adolescents: A cross-sectional study. BMC Health. 2011;11(247):1-10. doi: 10.1186/1471-2458-11-274. PubMed Central PMCID: PMCDec 5.

37. Carson V, Stone M, Faulkner G. Patterns of sedentary behavior and weight status among children. Pediatr Exerc Sci. 2014;26(1):95-102. doi: 10.1123/pes.2013-0061. PubMed PMID: 24092774.

38. Colley RC, Garriguet D, Janssen I, Wong SL, Saunders TJ, Carson V, et al. The association between accelerometer-measured patterns of sedentary time and health risk in children and youth: Results from the Canadian Health Measures Survey. BMC Public Health. 2013;13(200):9. doi: 10.1186/1471-2458-13-200.

39. Dorsey KB, Herrin J, Krumholz HM. Patterns of moderate and vigorous physical activity in obese and overweight compared with non-overweight children. Int J Pediatr Obes. 2011;6(2):E547-E55. doi: 10.3109/17477166.2010.490586. PubMed PMID: 20883127; PubMed Central PMCID: PMCPMC3815589.

40. Dowd KP, Harrington DM, Hannigan A, Donnelly AE. Light-intensity physical activity is associated with adiposity in adolescent females. Med Sci Sports Exerc. 2014;46(12):2295-300. doi: 10.1249/MSS.0000000000000357. PubMed PMID: 24797308.

41. Fletcher EA, Carson V, McNaughton SA, Dunstan DW, Healy GN, Salmon J. Does diet mediate associations of volume and bouts of sedentary time with cardiometabolic health indicators in adolescents? Obesity. 2017a;25(3):591-9. doi: 10.1002/oby.21750.

42. Fletcher EA, Lamb KE, McNaughton SA, Garnett SP, Dunstan DW, Baur LA, et al. Cross-sectional and prospective mediating effects of dietary intake on the relationship between sedentary behaviour and body mass index in adolescents. BMC Public Health. 2017b;17(1):1-10. doi: 10.1186/s12889-017-4771-0.

43. Garaulet M, Martinez-Nicolas A, Ruiz JR, Konstabel K, Labayen I, González-Gross M, et al. Fragmentation of daily rhythms associates with obesity and cardiorespiratory fitness in adolescents: The HELENA study. Clin Nutr. 2016. doi: 10.1016/j.clnu.2016.09.026. PubMed PMID: 27890490.

44. Holman RM, Carson V, Janssen I. Does the fractionalization of daily physical activity (sporadic vs. bouts) impact cardiometabolic risk factors in children and youth? PLoS One. 2011;6(10):7. doi: 10.1371/journal.pone.0025733. PubMed PMID: 21998688.

45. Mann KD, Howe LD, Basterfield L, Parkinson KN, Pearce MS, Reilly JK, et al. Longitudinal study of the associations between change in sedentary behavior and change in adiposity during childhood and adolescence: Gateshead Millennium Study. IJO. 2017;41(7):1042-7. doi: 10.1038/ijo.2017.69.

46. Mark AE, Janssen I. Influence of bouts of physical activity on overweight in youth. Am J Prev Med. 2009;36(5):416-21. doi: 10.1016/j.amepre.2009.01.027. PubMed PMID: 19362696.

47. Nettlefold L, McKay HA, Naylor PJ, Bredin SS, Warburton DE. The relationship between objectively measured physical activity, sedentary time, and vascular health in children. Am J Hypertens. 2012;25(8):914-9. doi: 10.1038/ajh.2012.68. PubMed PMID: 22673018.

48. Saunders TJ, Chaput JP, Goldfield GS, Colley RC, Kenny GP, Doucet E, et al. Prolonged sitting and markers of cardiometabolic disease risk in children and youth: A randomized crossover study. Metabolism. 2013a;62(10):1423-8. doi: 10.1016/j.metabol.2013.05.010. PubMed PMID: 23773981.

49. Thomas AS, Greene LF, Ard JD, Oster RA, Darnell BE, Gower BA. Physical activity may facilitate diabetes prevention in adolescents. Diabetes Care. 2009;32(1):9-13. doi: 10.2337/dc08-0780. PubMed PMID: 18840771; PubMed Central PMCID: PMCPMC2606821.

50. Fletcher EA, Salmon J, McNaughton SA, Orellana L, Wadley GD, Bruce C, et al. Effects of breaking up sitting on adolescents' postprandial glucose after consuming meals varying in energy: A cross-over randomised trial. J Sci Med Sport. 2017c:1-6. doi: 10.1016/j.jsams.2017.06.002.

51. Harrington SA. Relationships of objectively measured physical activity and sleep with BMI and academic outcomes in 8-year-old children. Appl Nurs Res. 2013;26(2):63-70. doi: 10.1016/j.apnr.2013.02.001. PubMed PMID: 23583266.

52. McManus AM, Ainslie PN, Green DJ, Simair RG, Smith K, Lewis N. Impact of prolonged sitting on vascular function in young girls. Exp Physiol. 2015;100(11):1379-87. doi: 10.1113/EP085355. PubMed PMID: 26370881.

53. Oliver M, Schluter PJ, Healy GN, Tautolo E, Schofield G, Rush E. Associations between breaks in sedentary time and body size in Pacific mothers and their children: Findings from the Pacific Islands Families Study. J Phys Act Health. 2013;10(8):1166-74.

54. Ross K, Hinckson E, Zinn C. Effect of intermittent sitting time on acute postprandial lipemia in children. J Clin Transl Endocrinol. 2015;2(2):72-6. doi: 10.1016/j.jcte.2015.03.003.

55. Stone MR, Rowlands AV, Middlebrooke AR, Jawis MN, Eston RG. The pattern of physical activity in relation to health outcomes in boys. Int J Pediatr Obes. 2009;4(4):306-15.

56. Blaes A, Baquet G, Fabre C, Van Praagh E, Berthoin S. Is there any relationship between physical activity level and patterns, and physical performance in children? Int J Behav Nutr Phys Act. 2011;8(122):1-8. doi: 10.1186/1479-5868-8-122. PubMed PMID: 22053790; PubMed Central PMCID: PMCDec 5.

57. Kwon S, Burns TL, Levy SM, Janz KF. Which contributes more to childhood adiposity-high levels of sedentarism or low levels of moderate-through-vigorous physical activity? The Iowa Bone Development Study. J Pediatr. 2013;162(6):1169-74. doi: 10.1016/j.jpeds.2012.11.071. PubMed PMID: 23305957; PubMed Central PMCID: PMCPMC3664130.

58. Willis EA, Ptomey LT, Szabo-Reed AN, Honas JJ, Lee J, Washburn RA, et al. Length of moderate-to-vigorous physical activity bouts and cardio-metabolic risk factors in elementary school children. Prev Med. 2015;73(1):76-80. doi: 10.1016/j.ypmed.2015.01.022. PubMed PMID: 25647532; PubMed Central PMCID: PMCPMC4455886.

59. Saunders TJ, Tremblay MS, Mathieu ME, Henderson M, O'Loughlin J, Tremblay A, et al. Associations of sedentary behavior, sedentary bouts and breaks in sedentary time with cardiometabolic risk in children with a family history of obesity. PLoS One. 2013b;8(11):e79143. doi: 10.1371/journal.pone.0079143. PubMed PMID: 24278117; PubMed Central PMCID: PMCPMC3835898.

60. Júdice PB, Silva AM, Berria J, Petroski EL, Ekelund U, Sardinha LB. Sedentary patterns, physical activity and health-related physical fitness in youth: A cross-sectional study. Int J Behav Nutr Phys Act. 2017;14(1). doi: 10.1186/s12966-017-0481-3.

75. Freedson P, Pober D, Janz KF. Calibration of Accelerometer Output for Children. Med Sci Sports Exerc. 2005;37(Supplement):S523-S30. doi: 10.1249/01.mss.0000185658.28284.ba (*reference not used in manuscript*).
