# Supplementary material for: Associations between activity patterns and cardio-metabolic risk factors in children and adolescents: A systematic review
Source: PLoS One. 2018 Aug 16;13(8):e0201947. doi: 10.1371/journal.pone.0201947 (PMC6095515; doi:10.1371/journal.pone.0201947)
Supplement: S5 Table — (PDF) [file pone.0201947.s005.pdf]

S5 Table. Studies reporting beneficial, non-significant and detrimental associations of activity patterns with inflammatory and endothelial blood biomarkers.

Moderate-to-Vigorous Physical Activity patterns						
**Time spent in bouts**	**Beneficial (B)**	**Non-significant (NS)**	**Detrimental (D)**	**B**	**NS**	**D**
**1-4 min**	C-reactive protein [44][A]			1	0	0
**1-9 min**	C-reactive protein [44][A]			1	0	0
**≥5 min**		C-reactive protein [44][A]		0	1	0
**≥10 min**	C-reactive protein [44][A]			1	0	0
Light Physical Activity patterns						
**Frequency of bouts**	**Beneficial (B)**	**Non-significant (NS)**	**Detrimental (D)**	**B**	**NS**	**D**
**≥4 s**	Acetylcholine[B] [55][C], [55][C]			2	0	0
**≥5 min**	Acetylcholine[B] [55][C], [55][C]			2	0	0
**Time spent in bouts**	**Beneficial (B)**	**Non-significant (NS)**	**Detrimental (D)**	**B**	**NS**	**D**
**≥4 s**		Acetylcholine[B] [55][C], [55][C]		0	2	0
**≥5 min**		Acetylcholine[B] [55][C], [55][C]		0	2	0

**Intensity of bouts**	**Beneficial (B)**	**Non-significant (NS)**	**Detrimental (D)**	**B**	**NS**	**D**
**≥4 s**		Acetylcholine[B] [55][C], [55][C]		0	2	0
**≥5 min**		Acetylcholine[B] [55][C], [55][C]		0	2	0
Sedentary patterns						
**Frequency of bouts/breaks**	**Beneficial (B)**	**Non-significant (NS)**	**Detrimental (D)**	**B**	**NS**	**D**
**1-4 min**		C-reactive protein [59][D], [59][E]		0	2	0
**5-9 min**		C-reactive protein [59][D], [59][E]		0	2	0
**5-10 min**		Adiponectin [14]; BDNF [14]; C-reactive protein [14]; IL2, 6, 8, 10[F] [14]; PAI-1 [14]; Resistin [14]; sE-selectin [14]; sICAM-1 [14]; sVCAM-1 [14]; TNF-α [14]		**0**	**13**	**0**
**10-14 min**		C-reactive protein [59][D], [59][E]		0	2	0
**15-29 min**	C-reactive protein [59][D]	C-reactive protein [59][E]		1	1	0
**≥10 min**		Adiponectin [14]; BDNF [14]; C-reactive protein [14]; IL2, 6, 8, 10[F] [14]; PAI-1 [14]; Resistin [14]; sE-selectin [14]; sICAM-1 [14]; sVCAM-1 [14]; TNF-α [14]		**0**	**13**	**0**
**≥30 min**		C-reactive protein [59][D], [59][E]		0	1	0

**Breaks**		Adiponectin [14]<br>BDNF [14]<br>C-reactive protein [14], [59][D], [59][E]; IL2, 6, 8, 10[F] [14]; PAI-1 [14]; Resistin [14]; sE-selectin [14]; sICAM-1 [14]; sVCAM-1 [14]; TNF-α [14]		**0**	**15**	**0**
**<u>Time spent in bouts/breaks</u>**	**Beneficial (B)**	**Non-significant (NS)**	**Detrimental (D)**	**B**	**NS**	**D**
**5-10 min**		Adiponectin [14]; BDNF [14]; C-reactive protein [14]; IL2, 6, 8, 10[F] [14]; PAI-1 [14]; Resistin [14]; sE-selectin [14]; sICAM-1 [14]; sVCAM-1 [14]; TNF-α [14]		**0**	**13**	**0**
**≥10 min**		Adiponectin [14]; BDNF [14]; C-reactive protein [14]; IL2, 6, 8, 10[F] [14]; PAI-1 [14]; Resistin [14]; sE-selectin [14]; sICAM-1 [14]; sVCAM-1 [14]; TNF-α [14]		**0**	**13**	**0**
**≥30 min**		C-reactive protein [36]		0	1	0
**Breaks**		C-reactive protein [36][G]		0	1	0

Abbreviations; *B* Beneficial, *D* Detrimental, *NS* Non significant, *min* Minutes, *s* Seconds, *BDNF* Brain-derived neurotrophic factor, *IL* interleukin, *PAI* Plasminogen Activator Inhibitor, *sICAM* Soluble Intercellular Adhesion Molecule, *sVCAM* Soluble Vascular Cell Adhesion Molecule, *TNF* Tumor Necrosis Factor.

[A] The sample was divided in four quartiles and the odds ratio of beneficial health factors were presented. When the odds consistently increased/decreased in all quartiles, we assumed that the associations were significantly beneficial/detrimental; [B] Two associations for Acetylcholine included in the table as both the absolute peak response and the

area under the flux versus time curve over the scans were reported in the study; [C] Only minimum borders for intensities were used to classify ≥light, ≥moderate, ≥vigorous and ≥very hard physical activity bouts; [D] Boys; [E] Girls; [F] All interleukin measures combined in table; [G] Percentage of time spent in intensity/percentage of sedentary time spent in breaks.

The bold numbers in the right hand columns tables represent that specific activity patterns which were examined at least four times.

References *(reference numbers correspond with manuscript)*

14. Gabel L, Ridgers ND, Della Gatta PA, Arundell L, Cerin E, Robinson S, et al. Associations of sedentary time patterns and TV viewing time with inflammatory and endothelial function biomarkers in children. Pediatr Obes. 2016;11(3):194-201. doi: 10.1111/ijpo.12045. PubMed PMID: 26097139.

33. Altenburg TM, de Niet M, Verloigne M, De Bourdeaudhuij I, Androutsos O, Manios Y, et al. Occurrence and duration of various operational definitions of sedentary bouts and cross-sectional associations with cardiometabolic health indicators: the ENERGY-project. Prev Med. 2015;71:101-6. doi: 10.1016/j.ypmed.2014.12.015. PubMed PMID: 25535676.

34. Bailey DP, Charman SJ, Ploetz T, Savory LA, Kerr CJ. Associations between prolonged sedentary time and breaks in sedentary time with cardiometabolic risk in 10–14-year-old children: The HAPPY study. J Sports Sci. 2017;35(22):2164-71. doi: 10.1080/02640414.2016.1260150.

35. Belcher BR, Berrigan D, Papachristopoulou A, Brady SM, Bernstein SB, Brychta RJ, et al. Effects of interrupting children's sedentary behaviors with activity on metabolic function: A randomized trial. J Clin Endocrinol Metab. 2015;100(10):3735-43. doi: 10.1210/jc.2015-2803. PubMed PMID: 26312582; PubMed Central PMCID: PMCPMC4596047.

36. Carson V, Janssen I. Volume, patterns, and types of sedentary behavior and cardio-metabolic health in children and adolescents: A cross-sectional study. BMC Health. 2011;11(247):1-10. doi: 10.1186/1471-2458-11-274. PubMed Central PMCID: PMCDec 5.

37. Carson V, Stone M, Faulkner G. Patterns of sedentary behavior and weight status among children. Pediatr Exerc Sci. 2014;26(1):95-102. doi: 10.1123/pes.2013-0061. PubMed PMID: 24092774.

38. Colley RC, Garriguet D, Janssen I, Wong SL, Saunders TJ, Carson V, et al. The association between accelerometer-measured patterns of sedentary time and health risk in children and youth: Results from the Canadian Health Measures Survey. BMC Public Health. 2013;13(200):9. doi: 10.1186/1471-2458-13-200.

39. Dorsey KB, Herrin J, Krumholz HM. Patterns of moderate and vigorous physical activity in obese and overweight compared with non-overweight children. Int J Pediatr Obes. 2011;6(2):E547-E55. doi: 10.3109/17477166.2010.490586. PubMed PMID: 20883127; PubMed Central PMCID: PMCPMC3815589.

40. Dowd KP, Harrington DM, Hannigan A, Donnelly AE. Light-intensity physical activity is associated with adiposity in adolescent females. Med Sci Sports Exerc. 2014;46(12):2295-300. doi: 10.1249/MSS.0000000000000357. PubMed PMID: 24797308.

41. Fletcher EA, Carson V, McNaughton SA, Dunstan DW, Healy GN, Salmon J. Does diet mediate associations of volume and bouts of sedentary time with cardiometabolic health indicators in adolescents? Obesity. 2017a;25(3):591-9. doi: 10.1002/oby.21750.

42. Fletcher EA, Lamb KE, McNaughton SA, Garnett SP, Dunstan DW, Baur LA, et al. Cross-sectional and prospective mediating effects of dietary intake on the relationship between sedentary behaviour and body mass index in adolescents. BMC Public Health. 2017b;17(1):1-10. doi: 10.1186/s12889-017-4771-0.

43. Garaulet M, Martinez-Nicolas A, Ruiz JR, Konstabel K, Labayen I, González-Gross M, et al. Fragmentation of daily rhythms associates with obesity and cardiorespiratory fitness in adolescents: The HELENA study. Clin Nutr. 2016. doi: 10.1016/j.clnu.2016.09.026. PubMed PMID: 27890490.

44. Holman RM, Carson V, Janssen I. Does the fractionalization of daily physical activity (sporadic vs. bouts) impact cardiometabolic risk factors in children and youth? PLoS One. 2011;6(10):7. doi: 10.1371/journal.pone.0025733. PubMed PMID: 21998688.

45. Mann KD, Howe LD, Basterfield L, Parkinson KN, Pearce MS, Reilly JK, et al. Longitudinal study of the associations between change in sedentary behavior and change in adiposity during childhood and adolescence: Gateshead Millennium Study. IJO. 2017;41(7):1042-7. doi: 10.1038/ijo.2017.69.

46. Mark AE, Janssen I. Influence of bouts of physical activity on overweight in youth. Am J Prev Med. 2009;36(5):416-21. doi: 10.1016/j.amepre.2009.01.027. PubMed PMID: 19362696.

47. Nettlefold L, McKay HA, Naylor PJ, Bredin SS, Warburton DE. The relationship between objectively measured physical activity, sedentary time, and vascular health in children. Am J Hypertens. 2012;25(8):914-9. doi: 10.1038/ajh.2012.68. PubMed PMID: 22673018.

48. Saunders TJ, Chaput JP, Goldfield GS, Colley RC, Kenny GP, Doucet E, et al. Prolonged sitting and markers of cardiometabolic disease risk in children and youth: A randomized crossover study. Metabolism. 2013a;62(10):1423-8. doi: 10.1016/j.metabol.2013.05.010. PubMed PMID: 23773981.

49. Thomas AS, Greene LF, Ard JD, Oster RA, Darnell BE, Gower BA. Physical activity may facilitate diabetes prevention in adolescents. Diabetes Care. 2009;32(1):9-13. doi: 10.2337/dc08-0780. PubMed PMID: 18840771; PubMed Central PMCID: PMCPMC2606821.

50. Fletcher EA, Salmon J, McNaughton SA, Orellana L, Wadley GD, Bruce C, et al. Effects of breaking up sitting on adolescents' postprandial glucose after consuming meals varying in energy: A cross-over randomised trial. J Sci Med Sport. 2017c:1-6. doi: 10.1016/j.jsams.2017.06.002.

51. Harrington SA. Relationships of objectively measured physical activity and sleep with BMI and academic outcomes in 8-year-old children. Appl Nurs Res. 2013;26(2):63-70. doi: 10.1016/j.apnr.2013.02.001. PubMed PMID: 23583266.

52. McManus AM, Ainslie PN, Green DJ, Simair RG, Smith K, Lewis N. Impact of prolonged sitting on vascular function in young girls. Exp Physiol. 2015;100(11):1379-87. doi: 10.1113/EP085355. PubMed PMID: 26370881.

53. Oliver M, Schluter PJ, Healy GN, Tautolo E, Schofield G, Rush E. Associations between breaks in sedentary time and body size in Pacific mothers and their children: Findings from the Pacific Islands Families Study. J Phys Act Health. 2013;10(8):1166-74.

54. Ross K, Hinckson E, Zinn C. Effect of intermittent sitting time on acute postprandial lipemia in children. J Clin Transl Endocrinol. 2015;2(2):72-6. doi: 10.1016/j.jcte.2015.03.003.

55. Stone MR, Rowlands AV, Middlebrooke AR, Jawis MN, Eston RG. The pattern of physical activity in relation to health outcomes in boys. Int J Pediatr Obes. 2009;4(4):306-15.

56. Blaes A, Baquet G, Fabre C, Van Praagh E, Berthoin S. Is there any relationship between physical activity level and patterns, and physical performance in children? Int J Behav Nutr Phys Act. 2011;8(122):1-8. doi: 10.1186/1479-5868-8-122. PubMed PMID: 22053790; PubMed Central PMCID: PMCDec 5.

57. Kwon S, Burns TL, Levy SM, Janz KF. Which contributes more to childhood adiposity-high levels of sedentarism or low levels of moderate-through-vigorous physical activity? The Iowa Bone Development Study. J Pediatr. 2013;162(6):1169-74. doi: 10.1016/j.jpeds.2012.11.071. PubMed PMID: 23305957; PubMed Central PMCID: PMCPMC3664130.

58. Willis EA, Ptomey LT, Szabo-Reed AN, Honas JJ, Lee J, Washburn RA, et al. Length of moderate-to-vigorous physical activity bouts and cardio-metabolic risk factors in elementary school children. Prev Med. 2015;73(1):76-80. doi: 10.1016/j.ypmed.2015.01.022. PubMed PMID: 25647532; PubMed Central PMCID: PMCPMC4455886.

59. Saunders TJ, Tremblay MS, Mathieu ME, Henderson M, O'Loughlin J, Tremblay A, et al. Associations of sedentary behavior, sedentary bouts and breaks in sedentary time with cardiometabolic risk in children with a family history of obesity. PLoS One. 2013b;8(11):e79143. doi: 10.1371/journal.pone.0079143. PubMed PMID: 24278117; PubMed Central PMCID: PMCPMC3835898.

60. Júdice PB, Silva AM, Berria J, Petroski EL, Ekelund U, Sardinha LB. Sedentary patterns, physical activity and health-related physical fitness in youth: A cross-sectional study. Int J Behav Nutr Phys Act. 2017;14(1). doi: 10.1186/s12966-017-0481-3.
