# Supplementary material for: Associations between activity patterns and cardio-metabolic risk factors in children and adolescents: A systematic review
Source: PLoS One. 2018 Aug 16;13(8):e0201947. doi: 10.1371/journal.pone.0201947 (PMC6095515; doi:10.1371/journal.pone.0201947)
Supplement: S7 Table — (PDF) [file pone.0201947.s007.pdf]

**S7 Table. Studies reporting beneficial, non-significant and detrimental associations of activity patterns with vascular health.**

| ***Moderate-to-Vigorous Physical Activity patterns*** | | | | | | |
|---|---|---|---|---|---|---|
| **Time spent in bouts** | **Beneficial (B)** | **Non-significant (NS)** | **Detrimental (D)** | **B** | **NS** | **D** |
| **0-5 min** | | Large artery compliance [47]; Small artery compliance [47] | | 0 | 2 | 0 |
| **1-4 min** | Systolic blood pressure [44][A] | | | 1 | 0 | 0 |
| **1-9 min** | Systolic blood pressure [44][A] | | | 1 | 0 | 0 |
| **≥5 min** | | Systolic blood pressure [44][A] | | 0 | 1 | 0 |
| **5-10 min** | | Large artery compliance [47]; Small artery compliance [47] | | 0 | 2 | 0 |
| **≥10 min** | | Systolic blood pressure [44][A] | | 0 | 1 | 0 |
| **10-20 min** | | Large artery compliance [47]; Small artery compliance [47] | | 0 | 2 | 0 |
| **≥20 min** | | Large artery compliance [47]; Small artery compliance [47] | | 0 | 2 | 0 |
| **Pattern types** | | Diastolic blood pressure 'Most vs. Sporadic', 'Most' vs. 'Medium', 'Medium' vs. 'Sporadic' [58][B]; Systolic blood pressure 'Most vs. Sporadic', 'Most' vs. 'Medium', 'Medium' vs. 'Sporadic' [58][B] | | **0** | **6** | **0** |

| *Sedentary patterns* | | | | | | |
|---|---|---|---|---|---|---|
| **Frequency of bouts/breaks** | **Beneficial (B)** | **Non-significant (NS)** | **Detrimental (D)** | **B** | **NS** | **D** |
| **Breaks** | | Diastolic blood pressure [34], [38][18/18]; Systolic blood pressure [34], [38][18/18] | | **0** | **38** | **0** |
| **≥20 min** | | Diastolic blood pressure [34]; Systolic blood pressure [34] | | 0 | 2 | 0 |
| **Time spent in bouts/breaks** | **Beneficial (B)** | **Non-significant (NS)** | **Detrimental (D)** | **B** | **NS** | **D** |
| **≥20 min** | | Diastolic blood pressure [38][18/18]; Systolic blood pressure [38][18/18] | | **0** | **36** | **0** |
| **≥30 min** | | Systolic blood pressure [36] | | 0 | 1 | 0 |
| **≥40 min** | | Diastolic blood pressure [38][18/18]; Systolic blood pressure [38][18/18] | | **0** | **36** | **0** |
| **≥60 min** | | Diastolic blood pressure [38][18/18]; Systolic blood pressure [38][18/18] | | **0** | **36** | **0** |
| **≥80 min** | | Diastolic blood pressure [38][18/18]; Systolic blood pressure [38][18/18] | | **0** | **36** | **0** |
| **≥100 min** | | Diastolic blood pressure [38][18/18]; Systolic blood pressure [38][18/18] | | **0** | **36** | **0** |
| **≥120 min** | | Diastolic blood pressure [38][18/18]; Systolic blood pressure [38][18/18] | | **0** | **36** | **0** |
| **Breaks** | | Systolic blood pressure [36][C] | | 0 | 1 | 0 |

| <u>**Duration of bouts/breaks**</u> | **Beneficial (B)** | **Non-significant (NS)** | **Detrimental (D)** | **B** | **NS** | **D** |
|---|---|---|---|---|---|---|
| **≥20 min** | | Diastolic blood pressure [34]; Systolic blood pressure [34] | | 0 | 2 | 0 |
| **Breaks** | | Diastolic blood pressure [34]; Systolic blood pressure [34] | | 0 | 2 | 0 |

Abbreviations; *B* Beneficial, *D* Detrimental, *NS* Non significant, *min* Minutes, *s* Seconds.

[A] The sample was divided in four quartiles and the odds ratio of beneficial health factors were presented. When the odds consistently increased/decreased in all quartiles, we assumed that the associations were significantly beneficial/detrimental; [B] Latent profile analyses divided sample in 'Sporadic', 'Medium', and 'Most bouts' pattern types. The percentage of MVPA accumulated in sporadic bouts (<5 min) was progressively lower, while the percentage MVPA in both short (5<10min) and medium-to-long bouts (≥10 min) was progressively higher moving from 'Sporadic', to 'Medium', and 'Most bouts'; [C] Percentage of time spent in intensity/percentage of sedentary time spent in breaks.

[X/18] Colley and colleagues reported associations between activity patterns and cardio-metabolic risk factors from 6 different subgroups (i.e. boys vs. girls in three different age groups; 6-10, 11-14, and 15-19 years) for 3 different time periods (e.g., after-school) [38]. X represents the number of associations categorised as beneficial, non-significant, or detrimental out of the total 18 associations tested.

The bold numbers in the right hand columns tables represent that specific activity patterns which were examined at least four times.

**References** *(reference numbers correspond with manuscript)*


14. Gabel L, Ridgers ND, Della Gatta PA, Arundell L, Cerin E, Robinson S, et al. Associations of sedentary time patterns and TV viewing time with inflammatory and endothelial function biomarkers in children. Pediatr Obes. 2016;11(3):194-201. doi: 10.1111/ijpo.12045. PubMed PMID: 26097139.

33. Altenburg TM, de Niet M, Verloigne M, De Bourdeaudhuij I, Androutsos O, Manios Y, et al. Occurrence and duration of various operational definitions of sedentary bouts and cross-sectional associations with cardiometabolic health indicators: the ENERGY-project. Prev Med. 2015;71:101-6. doi: 10.1016/j.ypmed.2014.12.015. PubMed PMID: 25535676.

34. Bailey DP, Charman SJ, Ploetz T, Savory LA, Kerr CJ. Associations between prolonged sedentary time and breaks in sedentary time with cardiometabolic risk in 10–14-year-old children: The HAPPY study. J Sports Sci. 2017;35(22):2164-71. doi: 10.1080/02640414.2016.1260150.

35. Belcher BR, Berrigan D, Papachristopoulou A, Brady SM, Bernstein SB, Brychta RJ, et al. Effects of interrupting children's sedentary behaviors with activity on metabolic function: A randomized trial. J Clin Endocrinol Metab. 2015;100(10):3735-43. doi: 10.1210/jc.2015-2803. PubMed PMID: 26312582; PubMed Central PMCID: PMCPMC4596047.

36. Carson V, Janssen I. Volume, patterns, and types of sedentary behavior and cardio-metabolic health in children and adolescents: A cross-sectional study. BMC Health. 2011;11(247):1-10. doi: 10.1186/1471-2458-11-274. PubMed Central PMCID: PMCDec 5.

37. Carson V, Stone M, Faulkner G. Patterns of sedentary behavior and weight status among children. Pediatr Exerc Sci. 2014;26(1):95-102. doi: 10.1123/pes.2013-0061. PubMed PMID: 24092774.

38. Colley RC, Garriguet D, Janssen I, Wong SL, Saunders TJ, Carson V, et al. The association between accelerometer-measured patterns of sedentary time and health risk in children and youth: Results from the Canadian Health Measures Survey. BMC Public Health. 2013;13(200):9. doi: 10.1186/1471-2458-13-200.

39. Dorsey KB, Herrin J, Krumholz HM. Patterns of moderate and vigorous physical activity in obese and overweight compared with non-overweight children. Int J Pediatr Obes. 2011;6(2):E547-E55. doi: 10.3109/17477166.2010.490586. PubMed PMID: 20883127; PubMed Central PMCID: PMCPMC3815589.

40. Dowd KP, Harrington DM, Hannigan A, Donnelly AE. Light-intensity physical activity is associated with adiposity in adolescent females. Med Sci Sports Exerc. 2014;46(12):2295-300. doi: 10.1249/MSS.0000000000000357. PubMed PMID: 24797308.

41. Fletcher EA, Carson V, McNaughton SA, Dunstan DW, Healy GN, Salmon J. Does diet mediate associations of volume and bouts of sedentary time with cardiometabolic health indicators in adolescents? Obesity. 2017a;25(3):591-9. doi: 10.1002/oby.21750.

42. Fletcher EA, Lamb KE, McNaughton SA, Garnett SP, Dunstan DW, Baur LA, et al. Cross-sectional and prospective mediating effects of dietary intake on the relationship between sedentary behaviour and body mass index in adolescents. BMC Public Health. 2017b;17(1):1-10. doi: 10.1186/s12889-017-4771-0.

43. Garaulet M, Martinez-Nicolas A, Ruiz JR, Konstabel K, Labayen I, González-Gross M, et al. Fragmentation of daily rhythms associates with obesity and cardiorespiratory fitness in adolescents: The HELENA study. Clin Nutr. 2016. doi: 10.1016/j.clnu.2016.09.026. PubMed PMID: 27890490.

44. Holman RM, Carson V, Janssen I. Does the fractionalization of daily physical activity (sporadic vs. bouts) impact cardiometabolic risk factors in children and youth? PLoS One. 2011;6(10):7. doi: 10.1371/journal.pone.0025733. PubMed PMID: 21998688.

45. Mann KD, Howe LD, Basterfield L, Parkinson KN, Pearce MS, Reilly JK, et al. Longitudinal study of the associations between change in sedentary behavior and change in adiposity during childhood and adolescence: Gateshead Millennium Study. IJO. 2017;41(7):1042-7. doi: 10.1038/ijo.2017.69.

46. Mark AE, Janssen I. Influence of bouts of physical activity on overweight in youth. Am J Prev Med. 2009;36(5):416-21. doi: 10.1016/j.amepre.2009.01.027. PubMed PMID: 19362696.

47. Nettlefold L, McKay HA, Naylor PJ, Bredin SS, Warburton DE. The relationship between objectively measured physical activity, sedentary time, and vascular health in children. Am J Hypertens. 2012;25(8):914-9. doi: 10.1038/ajh.2012.68. PubMed PMID: 22673018.

48. Saunders TJ, Chaput JP, Goldfield GS, Colley RC, Kenny GP, Doucet E, et al. Prolonged sitting and markers of cardiometabolic disease risk in children and youth: A randomized crossover study. Metabolism. 2013a;62(10):1423-8. doi: 10.1016/j.metabol.2013.05.010. PubMed PMID: 23773981.

49. Thomas AS, Greene LF, Ard JD, Oster RA, Darnell BE, Gower BA. Physical activity may facilitate diabetes prevention in adolescents. Diabetes Care. 2009;32(1):9-13. doi: 10.2337/dc08-0780. PubMed PMID: 18840771; PubMed Central PMCID: PMCPMC2606821.

50. Fletcher EA, Salmon J, McNaughton SA, Orellana L, Wadley GD, Bruce C, et al. Effects of breaking up sitting on adolescents' postprandial glucose after consuming meals varying in energy: A cross-over randomised trial. J Sci Med Sport. 2017c:1-6. doi: 10.1016/j.jsams.2017.06.002.

51. Harrington SA. Relationships of objectively measured physical activity and sleep with BMI and academic outcomes in 8-year-old children. Appl Nurs Res. 2013;26(2):63-70. doi: 10.1016/j.apnr.2013.02.001. PubMed PMID: 23583266.

52. McManus AM, Ainslie PN, Green DJ, Simair RG, Smith K, Lewis N. Impact of prolonged sitting on vascular function in young girls. Exp Physiol. 2015;100(11):1379-87. doi: 10.1113/EP085355. PubMed PMID: 26370881.

53. Oliver M, Schluter PJ, Healy GN, Tautolo E, Schofield G, Rush E. Associations between breaks in sedentary time and body size in Pacific mothers and their children: Findings from the Pacific Islands Families Study. J Phys Act Health. 2013;10(8):1166-74.

54. Ross K, Hinckson E, Zinn C. Effect of intermittent sitting time on acute postprandial lipemia in children. J Clin Transl Endocrinol. 2015;2(2):72-6. doi: 10.1016/j.jcte.2015.03.003.

55. Stone MR, Rowlands AV, Middlebrooke AR, Jawis MN, Eston RG. The pattern of physical activity in relation to health outcomes in boys. Int J Pediatr Obes. 2009;4(4):306-15.

56. Blaes A, Baquet G, Fabre C, Van Praagh E, Berthoin S. Is there any relationship between physical activity level and patterns, and physical performance in children? Int J Behav Nutr Phys Act. 2011;8(122):1-8. doi: 10.1186/1479-5868-8-122. PubMed PMID: 22053790; PubMed Central PMCID: PMCDec 5.

57. Kwon S, Burns TL, Levy SM, Janz KF. Which contributes more to childhood adiposity-high levels of sedentarism or low levels of moderate-through-vigorous physical activity? The Iowa Bone Development Study. J Pediatr. 2013;162(6):1169-74. doi: 10.1016/j.jpeds.2012.11.071. PubMed PMID: 23305957; PubMed Central PMCID: PMCPMC3664130.

58. Willis EA, Ptomey LT, Szabo-Reed AN, Honas JJ, Lee J, Washburn RA, et al. Length of moderate-to-vigorous physical activity bouts and cardio-metabolic risk factors in elementary school children. Prev Med. 2015;73(1):76-80. doi: 10.1016/j.ypmed.2015.01.022. PubMed PMID: 25647532; PubMed Central PMCID: PMCPMC4455886.

59. Saunders TJ, Tremblay MS, Mathieu ME, Henderson M, O'Loughlin J, Tremblay A, et al. Associations of sedentary behavior, sedentary bouts and breaks in sedentary time with cardiometabolic risk in children with a family history of obesity. PLoS One. 2013b;8(11):e79143. doi: 10.1371/journal.pone.0079143. PubMed PMID: 24278117; PubMed Central PMCID: PMCPMC3835898.

60. Júdice PB, Silva AM, Berria J, Petroski EL, Ekelund U, Sardinha LB. Sedentary patterns, physical activity and health-related physical fitness in youth: A cross-sectional study. Int J Behav Nutr Phys Act. 2017;14(1). doi: 10.1186/s12966-017-0481-3.
