# Supplementary material for: Associations between activity patterns and cardio-metabolic risk factors in children and adolescents: A systematic review
Source: PLoS One. 2018 Aug 16;13(8):e0201947. doi: 10.1371/journal.pone.0201947 (PMC6095515; doi:10.1371/journal.pone.0201947)
Supplement: S1 Text — (PDF) [file pone.0201947.s011.pdf]

S1 Text. Risk of bias assessment tool (based on items of the 'EPHPP Quality Assessment Tool for Quantitative Studies' [31]).

Selection bias

Q1: Are the individuals selected to participate in the study likely to be representative of the target population? (Very likely = 1; Somewhat likely = 2; Not likely = 3; Can't tell/Not reported = 4)

Are the participants/schools randomly selected? Is stated in the paper that the researchers attempted to create a sample representative of the target population?

Q2: What percentage of sampled individuals agreed to participate? (80 - 100% agreement = 1; 60-79% agreement = 2; less than 60% agreement = 3; Not applicable = 4; Can't tell/Not reported = 5)

What percentage of targeted individuals signed consent form?

Rate this section (Selection bias):	
STRONG	The selected individuals are very likely to be representative of the target population (Q1 is 1) and there is greater than 80% participation (Q2 is 1).
MODERATE	The selected individuals are at least somewhat likely to be representative of the target population (Q1 is 1 or 2); and there is 60 - 79% participation (Q2 is 2). 'Moderate' may also be assigned if Q1 is 1 or 2 and Q2 is 5 (can't tell).
WEAK	The selected individuals are not likely to be representative of the target population (Q1 is 3); or there is less than 60% participation (Q2 is 3) or selection is not described (Q1 is 4); and the level of participation is not described (Q2 is 5).

Confounders

Q3: Is there controlled for relevant confounders in either the design (e.g., stratification, matching) or analyses variates adjusted? (Most (80-100%) = 1; Some (60-79%) = 2; Few or none (Less than 60%) = 3; Can't tell/Not reported = 4)

Relevant confounders: Age, Clusters/Schools (when appropriate), Puberty, Sex, SES/Income etc., Wear time?

Rate this section (Confounders):	
STRONG	Will be assigned to those articles that controlled for at least 80% of relevant confounders (Q3 is 1).
MODERATE	Will be given to those studies that controlled for 60 – 79% of relevant confounders (Q3 is 2).
WEAK	Will be assigned when less than 60% of relevant confounders were controlled (Q3 is 3) or control of confounders was not described (Q3 = 4).

Data collection methods

Q4: Do the authors state in the article that valid measures of sedentary behaviour/physical activity are used? (Yes = 1; No = 2; Can't tell/Not reported = 3)

Have the device and chosen cut-points established validity in children or adolescents? Is the reference cited?

Q5: Do the authors state in the article that the measure of the sedentary behaviour/physical activity is reliable? (Yes = 1; No = 2; Can't tell/Not reported = 3)

Is the wear time (hours/day and total amount of days) reliable? Is there a reference provided for the inclusion criteria (e.g., hours chosen for valid day)?

Q6: Do the authors state in the article that valid measures of cardio-metabolic risk factor(s) are used? (Yes = 1; No = 2; Can't tell/Not reported = 3)

Have the data collection techniques established validity in children or adolescents? Is the reference cited? Do they state that a standardised approach has been used and reference this appropriately? Is there measured consistently using appropriate/standardised measures?

Q7: Was the measure of cardio-metabolic risk factor(s) reliable? (Yes = 1; No = 2; Can't tell/Not reported = 3)

Is the chosen technique reliable? Is there a reference provided?

Rate this section (Data collection methods):	
STRONG	The data collection tools have been shown to be valid (Q4 is 1 AND Q6 is 1); and the data collection tools have been shown to be reliable (Q5 is 1 AND Q7 is 1).
MODERATE	The data collection tools have been shown to be valid (Q4 is 1 AND 67 is 1); and the data collection tools have not been shown to be reliable (Q5 is 2 AND/OR Q7 is 2) or reliability is not described (Q5 is 3 AND/OR Q7 is 3)
WEAK	The data collection tools have not been shown to be valid (Q5 is 2 OR Q7 2) or both reliability and validity are not described (Q4 is 3 AND/OR Q6 is 3 and Q5 AND/OR Q7 is 3).

Withdrawals and drop-outs

Q8: Indicate the percentage of participants completing the objective assessment component of the study (80 -100% completion = 1; 60-79% completion = 2; less than 60% completion = 3; Can't tell/Not reported = 4; Not applicable = 5)

What percentage of the participants who have given consent completed the accelerometer measurements (to be included in the study)?

Rate this section (Withdrawals and drop-outs):	
STRONG	Will be assigned when the percentage of participants completing the study is 80% or greater (Q8 is 1).
MODERATE	Will be assigned when the percentage of participants completing the study is 60 – 79% (Q8 is 2) OR Q8 is 5 (N/A).
WEAK	Will be assigned the percentage of participants completing the study is less than 60% (Q8 is 3) or if the withdrawals and drop-outs were not described (Q8 is 4).

Global rating for this paper

Components that were not reported will be given a weak rating. Low risk of bias is classified as zero weak ratings for the 4 components, medium risk as 1 weak/4 ratings and high risk as ≥2 weak/4 ratings.

**Author, year**	**Selection bias**	**Confounders**	**Data collection methods**	**Withdrawals and dropouts**

Final decision of reviewer: 1 LOW ROB

2 MEDIUM ROB

3 HIGH ROB

References *(reference numbers correspond with manuscript)*

31. Effective Public Health Project. Quality Assessment Tool For Quantitative Studies [WWW document]. Hamilton, Ontario, Canada: Effective Public Health Project; 1998 [cited 2017 25 Jan]. Available from: http://www.ephpp.ca/tools.html.
